# Biology of Cardiac Troponins: Emphasis on Metabolism

**DOI:** 10.3390/biology11030429

**Published:** 2022-03-11

**Authors:** Aleksey M. Chaulin

**Affiliations:** 1 Department of Histology and Embryology, Samara State Medical University, 89 Chapaevskaya Street, Samara Region, 443099 Samara, Russia; a.m.chaulin@samsmu.ru; Tel.: +7-(927)-770-25-87; 2 Department of Cardiology and Cardiovascular Surgery, Samara State Medical University, 89 Chapaevskaya Street, Samara Region, 443099 Samara, Russia

**Keywords:** biology, cardiac troponins, cTns, metabolism, myocardial infarction, release mechanisms, proteolytic cleavage, elimination, diagnosis

## Abstract

**Simple Summary:**

Cardiovascular diseases, including myocardial infarction, are among the most common diseases worldwide. Markers associated with the diagnosis of myocardial infarction have been in the spotlight for many years. The most commonly used markers of myocardial infarction are cardiac troponins. However, insufficient understanding of the biology and metabolism of cardiac troponins does not allow us to fully unlock the full diagnostic potential of these cardiomarkers. In this article, I summarized and discussed in detail the features of the metabolism of cardiac troponins. I conducted a comprehensive review of current literary sources and presented my point of view. The format of the manuscript includes a consistent description of the biology and stages of the metabolism of cardiac troponins, starting from the release and circulation, and ending with the features of elimination of cardiac troponins. The possible influence of the biology of cardiac troponins on the diagnostic value of cardiac troponins is analyzed. Based on the analysis of the literature, I found a close relationship between the diagnostic value of cardiac troponins and their biology/metabolism. Further research is needed to increase the diagnostic value of cardiac troponins, and to fully unlock their diagnostic potential.

**Abstract:**

Understanding of the biology of endo- and exogenous molecules, in particular their metabolism, is not only of great theoretical importance, but also of high practical significance, since many molecules serve as drug targets or markers for the laboratory diagnostics of many human diseases. Thus, cardiac troponin (cTns) molecules have long been used as key markers for the confirmation of diagnosis of myocardial infarction (MI), and with the introduction of contemporary (high sensitivity) test methods, many of our concepts related to the biology of these cardiac markers have changed significantly. In current clinical practice, there are opening new promising diagnostic capabilities of cTns, the understanding and justification of which is closely connected with the theoretical principles of the metabolism of these molecules. However, today, the biology and metabolism of cTns have not been properly investigated; in particular, we do not know the precise mechanisms of release of these molecules from the myocardial cells (MCs) of healthy people and the mechanisms of circulation, and the elimination of cTns from the bloodstream. The main purpose of this manuscript is to systematize information about the biology of cTns, with an emphasis on the metabolism of cTns. The format of this paper, starting with the release of cTns in the blood and concluding with the metabolism/filtration of troponins, provides a comprehensive yet logically easy way for the readers to approach our current knowledge in the framework of understanding the basic mechanisms by which cTns are produced and processed. Conclusions. Based on the analysis of the current literature, the important role of biology and all stages of metabolism (release, circulation, removal) of cTns in laboratory diagnostics should be noted. It is necessary to continue studying the biology and metabolism of cTns, because this will improve the differential diagnosis of MI and i a new application of cTns immunoassays in current clinical practice.

## 1. Introduction

Troponins (troponin I (TnI), troponin T (TnT), troponin C (TnC)) are proteins that are parts of the troponin complex, which is bound to the protein tropomyosin (TPM). TPM, in its turn, together with actin, forms thin filaments of myocytes—the most important component of the contractile apparatus of striated muscle cells (of skeletal and cardiac muscles) [1,2]. All the three troponins participate in the calcium-dependent regulation of the striated muscle contraction–relaxation. Each troponin type fulfills specific regulatory functions in the contraction–relaxation of striated muscles. TnI is the inhibitory subunit of the TPM complex that binds actin during relaxation and inhibits the ATPase activity of actomyosin, thus preventing muscle contraction in the absence of calcium ions in the cell cytoplasm. TnT is the regulatory subunit, anchoring the troponin complex to thin filaments and, therefore, participating in the calcium-regulated contraction. TnC is the calcium-binding subunit. When the action potential is transferred to the muscle cell, calcium channels in the sarcoplasmic reticulum (“the repository of calcium ions”) open, and the sarcoplasmic reticulum releases calcium ions into the sarcoplasm. Then, calcium ions bind to TnC, which leads to conformational changes of proteins of the Tn-TPM complex, and as a result, the TPM molecule shifts and releases binding sites for the myosin head on the actin filament. It enables the interaction of the myosin head with actin, which underlies the mechanism of the contraction of striated muscles [3,4,5,6].

The molecules of troponins have different amino acid structures depending on their localization in muscles, on the basis of which troponin isoforms are distinguished. Thus, TnI has three isoforms: cardiac troponin I (cTnI), TnI of fast-twitch skeletal muscle fibers, and TnI of slow-twitch skeletal muscle fibers. TnT also has three main isoforms: cardiac troponin T (cTnT), the TnT of fast-twitch skeletal muscle fibers, and the TnT of slow-twitch skeletal muscle fibers. According to molecular genetic studies, the amino acid sequence of cTnI and cTnT differs from the amino acid sequences of the corresponding isoforms of skeletal Tns localized in skeletal muscle fibers by approximately 40–60% [7,8]. This important structural peculiarity allows for the use of cTnT and cTnI as specific biomarkers for the laboratory diagnostics of myocardial injury in MI and other non-cardiac pathological conditions. Cardiac troponin C (cTnC), as opposed to TnI and TnT, has a completely identical amino acid structure to the muscular (skeletal) TnC, and increased blood levels of this protein will not let us reliably distinguish the cardiac muscle tissue injury from the damage of skeletal muscles, and, therefore, cTnC cannot be used as a cardiac marker for MI diagnostics [9,10].

Notwithstanding the solid achievements in the study of etiopathogenesis, diagnostics, and treatment of acute coronary syndrome (ACS), it still remains one of the leading reasons for disability and mortality in all the developed countries of the world. All patients with ACS have a higher risk of developing myocardial infarction (MI) and death [11,12,13,14,15,16,17,18]. According to the results of the reviews of MI diagnostic criteria conducted in 2012 and 2018 by the European Society of Cardiology (ESC), the American College of Cardiology (ACC), the American Heart Association (AHA), and the World Heart Federation (WHF), the diagnosis verification is based on the presence of myocardial ischemia symptoms (clinical, electrocardiographic, echocardiographic, angiographic) and the positive dynamics of cTns levels in the blood of patients [14,15,16,17,18].

**Figure 1:**
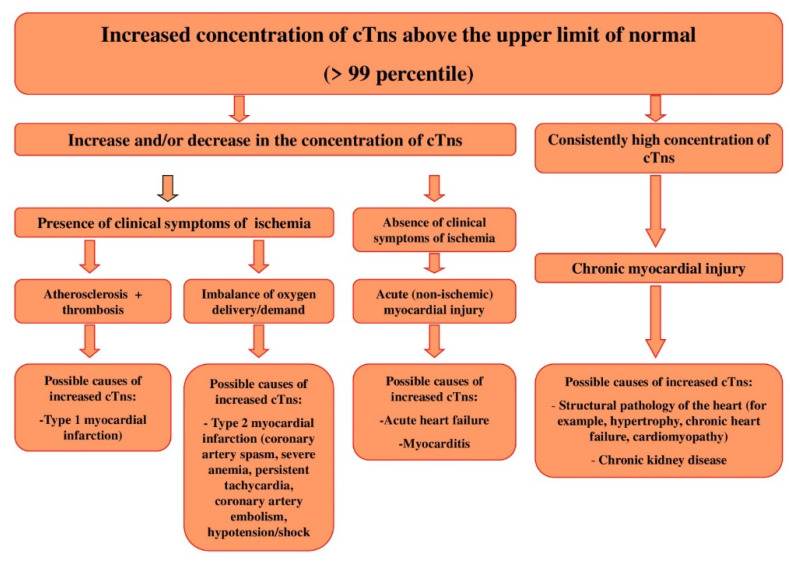
Interpretation of possible reasons for myocardial injury and increase in cTns serum levels.

Although the regulatory documents concerning the diagnostics and treatment of different forms of ACS and MI contain clear recommendations on the time of troponin testing and decision-making levels, and the sensitivity and specificity of most immunoassays approximate 100%, there still remain a number of unsolved problems and issues relating to the application of these markers in clinical practice. Some of these problems are connected with the variety of troponin diagnostic agents, their unequal sensitivity and diagnostic accuracy, and different susceptibility to cross-reactive substances (molecules), i.e., with different analytical characteristics of test systems [19,20]. Another range of issues results from the fact that the increase in cTns levels takes place in the case of myocardial necrosis of any etiology, and sometimes in the absence of irreversible myocardial injury (for instance, in case of reversible injury induced by physical exercises, renal failure, or the influence of false-positive factors) [21,22,23,24,25,26,27,28]. Besides, along with the necrosis of cardiomyocytes, there are other mechanisms of cardiac troponin release from myocardial cells (MCs) and/or the increase in cTns concentration in blood serum. Thus, several clinical studies provide evidence of a very frequent increase in cardiac troponin concentration in various pathologies [29,30,31,32]. At the same time, the mechanism of troponin increase in these diseases is not associated with the ischemic necrosis of MCs—the main mechanism of troponin levels increases in MI. The study by G. Lindner et al. is quite representative in this respect. The researchers have conducted a detailed analysis of the reasons (the diseases) for an increase in cTnT levels in patients admitted to the emergency department. In total, the study included 1573 patients, only 10% of which had an increased level of cTnT associated with MI, while all the rest (about 90%) showed no signs of MI, and their increased levels of cTnT were induced by other diseases, leading to an increase in cTnT serum levels via non-ischemic mechanisms. The most common reasons for cTnT increase were the following: pulmonary embolism, renal failure, acute aortic dissection, heart failure, acute myocarditis, rhabdomyolysis, the application of cardiotoxic chemotherapeutic agents, the acute exacerbation of chronic obstructive pulmonary disease, sepsis, and infiltrative cardiac pathologies (for example, amyloidosis). The interesting fact revealed by this study was that, in 30% of cases, increased levels in cTnT were not connected with any previously described causes of cardiac troponin increase [29]. There is a high probability that these reasons might be connected to the false-positive mechanisms, or they have been induced by factors that the researchers and medical practitioners have not paid attention to, and have not described yet. Thus, the interpretation of the results with increased levels of cTns is an extremely complicated and sometimes even impossible task of modern clinical practice. Therefore, it is important to remember that the troponin test itself is not “the gold standard test” for MI diagnostics, but it can become one only for those patients who show typical clinical symptoms of myocardial ischemia, and have corresponding ischemic changes on the electrocardiogram, echocardiogram, etc. Generally, when interpreting possible reasons for the increase in cTns in blood serum, one should be guided by the following schematics (Figure 1).

Due to modern ultra-sensitive tests, medical practitioners have the opportunity to diagnose MI early (within the first two hours from the admission of the patient) through the evaluation of the dynamic changes of cTns. The changes (increases) in the concentration of cardiac troponin molecules within the first two hours are very small (may amount to as little as several ng/L), and cannot be detected by moderately sensitive test systems. It should be noted that, due to a number of multicenter studies, algorithms of the early diagnostics (0 → 1 h, and 0 → 2 h) of non-ST-segment elevation MI (NSTEMI) have been validated for the ultra-sensitive test systems of various manufacturers (Table 1) [33].

According to the data of modern (ultra-sensitive) troponin test methods, the molecules of cTns are detected in blood and a number of other biological fluids in all healthy people [34,35,36,37,38,39,40,41,42], which poses new issues and challenges for scientists when it comes to the search for, and explanation of, possible mechanisms underlying the release of troponin molecules from intact MCs. Hence, the molecules of cTns can be considered normal products of cardiac muscle tissue metabolism. However, the precise mechanisms of release are not clear yet, and are hypothetical. Moreover, the factors that may influence and facilitate or, on the contrary, reduce the release of cTns will be of great importance for researchers and medical practitioners. Currently, the most discussable biological factors influencing the degree of troponin release from healthy myocardium are gender, age, and circadian characteristics [43,44,45,46,47,48,49,50,51,52]. The gender-related characteristics of cTns consist of the fact that the myocardium of healthy men releases more molecules of cTns than that of healthy women. These characteristics are validated by many clinical studies, and practically in all modern test systems, it is recommended to use the threshold values (99th percentile) in accordance with gender [45,53,54]. The age-related characteristics of cTns are that a greater amount of cTns molecules are released from the myocardium of elderly patients compared to the myocardium of young people [49,55,56]. The circadian features of cTns are that more cardiac troponin molecules are released from the myocardium of healthy people in the morning than from the myocardium of healthy people in the evening–night period [57,58]. It should be noted that the age and circadian characteristics of cTns are not typical for all test systems, and according to some studies, they are contradictory [59,60,61]. Before using these characteristics in rapid diagnostic algorithms, it is necessary to conduct additional large studies to validate the age and circadian characteristics of cTns.

One of the significant problems of both moderately sensitive and modern highly sensitive immunoassays is the lack of their standardization [62,63,64]. This leads to the fact that different troponin immunoassays detect different values (concentrations) of cardiac troponin molecules in the blood and other biological fluids of the same patient. So, in accordance with the data in Table 1, the threshold levels for excluding/confirming NSTEMI differ by several degrees when using immunoassays from different manufacturers [33]. Based on this, we can say that each method detects, in fact, the different molecules of cTns and their fragments in a biological fluid. This creates certain difficulties and problems: (1) the need to validate the threshold concentrations of cTns for each test system, including newly developed ones, which is associated with additional high costs; (2) the need for a thorough study of interfering factors for each of the known detection methods that also involves additional costs; (3) upon admission of a patient to the hospital, dynamic changes in cTns to confirm/exclude MI during the first and subsequent hours can be traced and evaluated only when using the same test system, and immunoassays from different manufacturers cannot be used for this goal. At the same time, different institutions can use different test systems, which will not allow for a proper assessment of the dynamic changes in the case of the urgently required transportation of a patient to another institution, which will be associated with a loss of time for additional examinations and additional economic costs.

It should be noted separately that the molecules of cTns can be affected by the very numerous proteolytic enzymes present in the blood, which thereby can indirectly affect the levels of cTns in the blood of patients. For example, due to an increase in the activity of proteases that cause the fragmentation of troponin molecules, the duration of the circulation (half-life) of cTns in the bloodstream will decrease, which can potentially lead to false-negative results when using those test systems that have diagnostic antibodies directed against the fragmented epitopes of troponin molecules [65,66]. A decrease in the activity of such proteolytic enzymes, on the contrary, may hypothetically lead to false-positive results. However, current specific knowledge about this metabolic stage, in particular, exact information about all the influencing enzymes and mechanisms of troponin fragmentation, is extremely scarce, and is not taken into account in clinical practice. This stage of troponin metabolism and its effect on diagnostics will be considered in more detail in this manuscript below in the paragraph on the circulation of cTns in blood plasma.

A very interesting direction in studying the diagnostic value of cTns is the assessment of the possibility of using other biological fluids as biomaterials for the detection of troponin molecules. This direction is developing due to an increase in the sensitivity of immunoassays (the creation of highly sensitive and ultra-sensitive test systems), which can detect very low concentrations (at levels of several ng/L) of cTns that circulate in many biological fluids, including non-invasively obtained fluids (urine and oral fluid) [38,39,40,41,42,43,44,45,46,47,48,49,50,51,67,68,69,70,71,72,73]. Moderately sensitive methods, as a rule, cannot detect such low concentrations of cardiac troponin molecules present in these biological fluids [74,75]. The mechanisms of the penetration/transport of cTns into these biological fluids should also be considered as one of the stages of the metabolic pathway of cTns. Moreover, the study and understanding of precise mechanisms of penetration/transport of cTns will increase their diagnostic value and validate new methods for diagnosing cardiovascular diseases through the use of other biological fluids; in particular, non-invasively obtained fluids, since their collection has a number of advantages (for example, painlessness and atraumatic nature, lower risk of introduction of blood-borne infections, and the possibility of obtaining biomaterial without the involvement of medical personnel) over the use of blood as a biomaterial. In addition, there are prospects for the creation of specialized diagnostic test strips (“dry chemistry” methods) for the detection of cTns in urine and/or oral fluid, which will make it possible to carry out the express diagnostics and/or monitoring of cardiovascular diseases at home by patients themselves, or by their relatives.

The main biological fluids in which the molecules of cTns are detected and their diagnostic value are summarized in Table 2.

## 2. Metabolic Pathway of cTns

**Figure 2:**
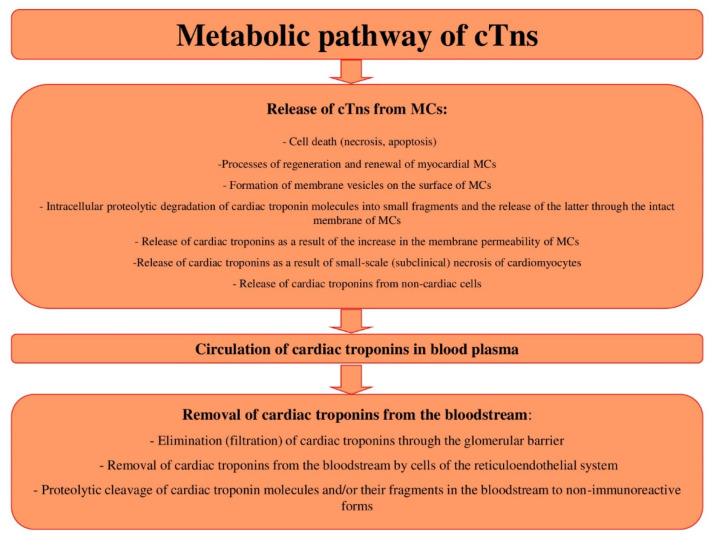
Metabolic pathway of cTns.

Conventionally, three main stages of the metabolic pathway of cTns can be distinguished (Figure 2): (1) the release of cTns from MCs, (2) the circulation of cTns in blood plasma, (3) the removal of cTns from the bloodstream. Similar key stages of the metabolism of molecules are distinguished for other molecules, in order to conveniently and consistently consider the main metabolic characteristics of molecules.

Each of these stages of metabolism can play a decisive role in the regulation of the concentrations of cTns in blood, i.e., their diagnostic value. In addition, there are a number of factors that can have a potential and hypothetical influence on these stages of the metabolic pathway of cTns. These factors can be physiological conditions; for example, gender, age, and circadian characteristics, which have a certain effect on the degree of release of troponin molecules from the myocardium of healthy people, or changes in the activity of the proteolytic enzymes that target cardiac troponin molecules. The activity of proteolytic enzymes can also change under pathological conditions and/or in the case of taking certain medications. Renal failure can be noted as an example of a significant factor affecting the removal of cTns from the bloodstream. It is important to emphasize that there may be an extremely large number of such factors, and some of them are probably still unknown.

Furthermore, in the course of this manuscript, I will sequentially consider each of the stages of the metabolic pathway of cTns, and note the main known and assumed factors that may affect these mechanisms and the diagnostic value of cTns.

### 2.1. Release of cTns from MCs: Mechanisms and Diagnostic Value

The introduction of highly sensitive test systems into practice made it possible, with high accuracy (variability of the analysis within 10%), to detect very low concentrations of cTns, ranging from 0.001 to 0.01 ng/mL, and below the values corresponding to the 99th percentile (upper limit of the norm). As a result, cTns molecules were found in almost 100% of healthy people, and instead of a clear borderline level typical of MI, a smooth scale appeared, capable of reflecting the subclinical myocardial pathology associated with structural (non-ischemic) damage, stable coronary artery diseases, and other pathological conditions that negatively affect MCs [87,88,89]. Considering the fact that the molecules of cTns began to be detected in all healthy people, it became necessary to study and explain the mechanisms of the release of cTns from the intact myocardium. In this regard, the researchers are discussing the following possible mechanisms for the release of cTns molecules, and an increase in their serum levels: (a) the release of cTns as a result of the processes of regeneration and renewal of MCs, (b) the release of cTns as a result of the apoptosis of MCs, (c) the release of cTns as a result of the formation of membrane vesicles on the surface of MCs, (d) the intracellular proteolytic degradation of cardiac troponin molecules into small fragments, and the release of the latter through the intact membrane of MCs, (e) the release of cTns as a result of the increase in the membrane permeability of MCs, (f) the release of cTns as a result of the small-scale (subclinical) necrosis of cardiomyocytes, (g) the release of cTns from non-cardiac cells. Some of the above mechanisms of release can not only explain the detectable concentrations of cTns in healthy individuals, but also significantly activate/increase under certain physiological conditions and pathological processes [90,91,92,93]. For example, the apoptosis of MCs can increase with an increase in blood pressure [94,95], the stretching of the myocardial walls [96,97], the increased stimulation of beta-adrenergic receptors [98,99,100], and a number of other mechanisms [101,102], which thereby may facilitate the release of cTns from cardiomyocytes. Furthermore, in conditions of chronic renal failure, the expression of cTns in skeletal muscles is noted [103], which, according to some authors, can lead to an increase in the serum concentrations of cTns in patients with chronic renal failure [103,104].

Below, I will consider each of the above mechanisms of cTns release, sequentially and in more detail.

### 2.2. Release of cTns as a Result of the Processes of Regeneration and Renewal of MCs

Evidence of the fact that MCs can regenerate/renew has been obtained by studying the metabolism of C^14^-labeled DNA molecules in MCs. A long-term observation of people with the inclusion of a radioactive isotope of carbon (C^14^) in the DNA of cardiomyocytes, which occurred as a result of nuclear weapons tests, was carried out. The authors calculated the rate of renewal of cardiomyocytes by studying the rate of DNA synthesis, which was calculated by investigating the rate of accumulation of C^14^ in MCs. A renewal of cardiomyocytes, the intensity of which decreased annually—from 1% per year at the age of 25 years to 0.45% per year at the age of 75 years—was found. In general, about 50% of cardiomyocytes underwent renewal throughout life [105,106]. These results indicate the existence of a small regenerative potential in MCs. Some researchers suggest that the process of renewal of MCs may be associated with the release of cTns from cells [107], however, the specific mechanism underlying this phenomenon remains unknown. As a possible hypothesis, it can be assumed that the intracellular molecules of cardiac markers, including cTns, will be released from gradually aging and naturally dying cardiomyocytes, as a result of the gradual destruction of the cell membrane. Since the rate of renewal of cardiomyocytes is low, the degree of increase in the serum levels of cTns is also insignificant (no higher than the 99th percentile). Thus, this mechanism can hypothetically explain the presence of a small amount of cTns molecules in the bloodstream of all healthy individuals.

In accordance with the data of other researchers, the average rate of cardiomyocyte renewal in mammals is 0.5–2.0% per year, and can vary depending on the influence of certain factors, such as physiological conditions (physical activity), trauma, and concomitant diseases [108,109,110]. Thus, the rate of renewal of cardiomyocytes increases significantly after myocardial damage. An experimental study conducted by Docshin et al. has shown that ischemic myocardial injury causes the activation of endogenous stem cells, and increases the rate of myocardial cell renewal [111]. Two other research groups led by Waring et al. [112] and Rovira et al. [113,114] revealed an increase in the processes of proliferation and differentiation of stem cells in the myocardium of rats and zebrafish.

However, the assessment of the degree of regeneration and the rate of renewal of MCs can be significantly influenced by inflammatory processes, the proliferation of non-myocyte cells, and the formation of a connective tissue scar in the myocardium, which often complicates and/or distorts investigation results [115,116]. Further research is needed to investigate the specific role of myocardial cell regeneration and renewal in the release of cTns from cells.

### 2.3. Release of cTns as a Result of Apoptosis of MCs

To date, a large number of factors have been discovered that can trigger the processes of the apoptosis of cardiomyocytes [117,118]. The induction of apoptosis leads to an increase in the activity of caspases (proteolytic enzymes of the cysteine protease family), which can fragment (damage) DNA and protein molecules, leading to cell death. In contrast with necrosis, during apoptosis, the cell dies more slowly, the integrity of the cell membrane remains much longer, and the inflammatory reaction around the dead cell is not observed. To study the processes of apoptosis, many methods are used: various types of microscopy (light, electron, fluorescence), flow cytometry, immunohistochemical analysis, the TUNEL method (terminal deoxynucleotidyl transferase (TdT) dUTP nick-end labeling), etc. The TUNEL method is the most reliable and early method for detecting apoptosis. This method permits the visualization of cell nuclei in which the DNA molecule has been fragmented, due to the increased activity of endonucleases and caspases. This method is most often used in all modern studies aimed at the exploration of the etiopathogenetic mechanisms of the apoptosis of various cells, including cardiomyocytes [119,120,121].

An experimental study led by Weil et al. has shown that the short-term ischemia activates apoptosis of MCs in experimental animals, and the apoptosis of cardiomyocytes is accompanied by an increase in the serum levels of cTns. Short-term myocardial ischemia was simulated by means of the balloon occlusion of a branch of the left coronary artery, and the fact of occlusion was confirmed by coronary angiography. The duration of ischemia was 10 min, after which reperfusion was carried out by deflation of the balloon. To confirm the apoptosis of MCs, the TUNEL method was used, according to the results of which, the number of cardiomyocytes in the state of apoptosis was significantly increased (six times as many as the control group of animals). At the same time, no histological signs of myocardial necrosis were observed. This suggests that short-term (in this case, 10-min) ischemia does not cause the ischemic necrosis of cardiomyocytes, but enhances apoptotic processes in the myocardium. However, the levels of cTns began to rise rapidly: 30 min after reperfusion, the cTnI concentration approached the upper limit of the norm (38 ng/L), and after 1 h, exceeded it (51 ± 17 ng/L). Two and three hours after reperfusion, the serum levels of cTnI were 148 ± 88 ng/L and 180 ± 117 ng/L, respectively, which indicated the continuing release of troponin molecules from the myocardium. Finally, 24 h after reperfusion, the cTnI concentration reached its peak and amounted to 1021 ± 574 ng/L [122]. Thus, this experimental study elegantly demonstrates the role of apoptosis (induced by short-term ischemia) in the release of cardiac troponin molecules from MCs. The limitation of this study is the relatively short interval of investigating the cardiac muscle tissue for the presence of histopathological changes; according to these results, it is impossible to determine the degree and reversibility of damage to cardiac myocytes during the apoptosis induced by short-term ischemia. Besides, for the detection of cTnI, a moderately sensitive test system was used, which is inferior in diagnostic capabilities to modern highly sensitive immunoassays. In this regard, dynamic changes in the levels of cTns detected by highly sensitive immunoassays during apoptosis could be significantly different, especially in the first minutes and hours after reperfusion.

The literature also describes other situations when the apoptosis of cardiac myocytes is induced by other mechanisms that are not associated with the reversible (short-term) ischemia of cardiac muscle tissue. The authors identify the following mechanisms of apoptosis that can promote the release of cTns from MCs: the stretching of the myocardial walls, increased preload on the heart, and the increased activity of the sympathoadrenal system [94,95,96,97,98,99,100,123]. Thus, Cheng et al. reported that the apoptosis of MCs increases with the stretching of the myocardial walls [123]. This allows us to consider the many physiological and pathological conditions that cause the stretching of the myocardial walls as possible inducers of apoptosis, and thus can, to some extent, explain the increased serum levels in patients after prolonged and intense physical exertion, or having arterial hypertension, pulmonary embolism, chronic obstructive pulmonary disease, and a number of other pathologies [123,124,125,126].

In another experimental study, Weil et al. concluded that an increase in preload on the heart triggers apoptosis, and causes an increase in the concentration of cTnI in the blood of experimental animals. To increase the preload on the heart, the experimental group of animals received an intravenous drug—phenylephrine (300 μg of the drug per minute)—for one hour. After the simulation, echocardiography was used to confirm myocardial overload, and to verify apoptosis and determine cardiac troponin levels, histological methods were used, including the TUNEL method and a moderately sensitive immunoassay, respectively. As a result of the histological examination of the myocardium of the experimental group of animals, a significant increase in the number of cardiomyocytes in the state of apoptosis was noted, as compared to the control group. At the same time, no histological signs of myocardial necrosis were recorded. Moreover, 24 h after the simulation, the number of cardiomyocytes in the state of apoptosis decreased to the level of the control group, which indicates the reversibility of apoptotic changes. The cTnI concentration exceeded the upper limit of the norm 30 min after the simulation, and then the cTnI levels continued to rise sharply, and reached a value of 856 ± 956 ng/L one hour after the simulation. The serum levels of cTnI remained elevated throughout the study period (24 h) and peaked at 1462 ± 1691 ng/L [127]. Since signs of necrosis, unlike apoptosis, were not observed, it should be considered that apoptosis induced by myocardial overload plays an important role in the release of cardiac troponin molecules from cells [128].

The degree of release of cardiac troponin molecules from MCs as a result of apoptosis induced by myocardial overload depends on the strength and duration of exposure. For example, relatively small myocardial overload is observed on mild to moderate exertion, in hypertension and non-massive pulmonary embolism, so the increase in serum levels of cTns in these conditions is also relatively small. However, for example, on high-intensity exertion, or in massive pulmonary embolism, myocardial overload becomes much more significant, and therefore, these conditions are accompanied by relatively higher serum levels of cTns [129,130,131].

Another very interesting mechanism for the initiation of apoptosis is an increase in the activity of the sympathoadrenal system. A research group led by Singh et al. found that the stimulation of beta-adrenergic receptors (β-AR) regulates intracellular apoptotic signaling pathways in cardiomyocytes. Moreover, the stimulation of β1-AR enhances the apoptosis of MCs, while the stimulation of β2-AR has the opposite effect [132,133]. It was also noted that the density of β-AR subtypes changes significantly with age [134]. Thus, in elderly patients, a more pronounced decrease in the number of β2-AR is noted, which may contribute to a weakening of the anti-apoptotic effect and, accordingly, an increase in the apoptosis of MCs [134,135,136]. A higher degree of apoptosis in elderly patients can probably be associated with the age-related characteristics of cardiac troponin levels: in older people, troponin levels are significantly higher than in young people. The age-related characteristics of cTns have been demonstrated in a number of clinical studies through blood examination with highly sensitive test systems [46,47,48,49,55,56].

Considering the above, the apoptosis of cardiomyocytes should be considered as a significant mechanism for the release of cardiac troponin molecules from MCs. This mechanism is not associated with the necrosis of cardiomyocytes, and contributes to a very significant increase in the serum levels of cTns. Thus, the apoptosis of MCs can be of great diagnostic value in conditions such as prolonged and intense physical activity, arterial hypertension, pulmonary embolism, heart failure, and, probably, in old age. Further research is needed to clarify the exact role of apoptosis in the release of cTns in physiological and pathological conditions.

### 2.4. Release of cTns as a Result of the Formation of Membrane Vesicles on the Surface of MCs

This mechanism of release of cTns from MCs was first described in an experimental study a relatively long time ago. A research group led by Schwartz reported that, on the plasma membrane of MCs, membrane vesicles (blebbing vesicles) are formed [137,138]. Moreover, during ischemia, the number of blebbing vesicles increases in comparison with intact MCs. A similar trend is also typical for hepatocytes [139]. Since these vesicles are formed from fragments of the cell membrane and the cytoplasm of cardiomyocytes, these vesicles may contain some cytoplasmic proteins of MCs; in particular, cardiac markers (creatine kinase MB isoform, myoglobin, and the cytoplasmic fraction of cTns, and others). However, since the volume of the cytoplasmic fraction of cTns is small (approximately 3–4% for cTnI and 7–8% for cTnT of the total amount of cTns in the cardiomyocyte) [57,140], the contribution of this mechanism to the degree of increase in serum levels of cTns will also be limited. Based on the peculiarities of the formation of blebbing vesicles (a significant increase in ischemia), it can be assumed that this mechanism is involved in the release of cTns in those pathological conditions that are accompanied by the ischemia of MCs at an early stage. For example, the initial (prenecrotic) stage of myocardial ischemia can provoke the formation of blebbing vesicles, and the release of cTns into the bloodstream, which will lead to the formation of the first peak in serum concentrations of cTns. Subsequently, two main scenarios are possible: (1) with a decrease of the ischemia of MCs, the formation of blebbing vesicles stops, and troponin concentrations quickly return to normal, (2) with the continuation/intensification of ischemia (as, for example, during MI), the formation of blebbing vesicles increases, and, in addition to this, the destruction of the plasma membrane of MCs and proteolysis (fragmentation) of troponin proteins, which are part of the main (structural or contractile) troponin fraction, occurs, which will lead to the formation of a second peak in the serum concentrations of cTns. From a pathogenetic point of view, any physiological or pathological condition that will lead to the ischemia of MCs (even reversible myocardial ischemia) can activate this mechanism of cardiac troponin release. For instance, some physiological conditions (physical activity) [141] or pathological conditions (sepsis) [142] can cause an increase in the oxygen demand of MCs, which, accordingly, will be accompanied by ischemia of the cardiac muscle tissue.

### 2.5. Intracellular Proteolytic Degradation of cTns Molecules into Small Fragments and the Release of the Latter through the Intact Membrane of MCs

The size and location of intracellular molecules are two key factors that affect the transport (release) of molecules across the cell membrane. Low molecular weight biomarkers are much more intensively released across the plasma membrane, which plays a role in the diagnostics of many diseases, including cardiovascular pathologies. So, for example, with the development of MI, the concentration of low molecular weight cardiac markers (myoglobin) in blood serum increases much earlier than the concentration of high molecular weight cardiac markers (lactate dehydrogenase-1) [143,144,145]. This is due to the fact that myoglobin molecules are small and can be released at the initial stages of ischemia during the development of MI (when the plasma membrane of MCs is still relatively insignificantly damaged). A larger molecule (lactate dehydrogenase-1) can leave the cardiomyocyte only when its cell membrane is significantly damaged. Biomarkers that are freely localized in the cytoplasm of cells (for example, myoglobin, the cytoplasmic (non-contractile) fraction of cTns) also have advantages when released from the cell, in contrast to those biomarkers that are localized in organelles (nucleus or mitochondria of cells) or which are tightly bound to structural components of the sarcoplasm (for example, the structural fraction of cTns involved in the regulation of the contractile fraction of the myocardium). So, during the development of MI, the primarily released molecules are troponin molecules that are part of the cytoplasmic fraction of cTns, and only then does the destruction of sarcomeres take place (in particular of the Tn-TPM complex, and the release of structural cTns).

The most important factor that can affect the size of a molecule (biomarker) and, accordingly, the possibility of its release, is the degree of activity of enzymes that cause the proteolysis (fragmentation) of this molecule [146,147]. The activity of proteolytic enzymes can change both under physiological and pathological conditions. In an experimental study conducted by Feng et al., it was demonstrated that an increase in preload on cardiac muscle tissue activates the enzyme calpain, which fragments the cTnI molecule, which could potentially play a role in the release of this biomarker from MCs and an increase in its level in blood serum [147]. Thus, physiological and pathological conditions causing an increase in the preload on the myocardial wall can promote the release of cTns from MCs by this mechanism.

In addition to the enzyme calpain, the cleavage of cTns molecules can be catalyzed by some types of matrix metalloproteinases (MMP 2 and MMP 14) [147,148,149,150] and the enzyme thrombin [151,152,153]. The activity of these enzymes can also be influenced by pathological processes and some drugs, which thereby, hypothetically, can affect the serum levels of cTns. For example, an increase of thrombin activity in patients with dilated cardiomyopathy [154,155] can contribute to the fragmentation of cTnT, which can have both pathogenetic significance (damage to cTnT, which is one of the main components of the contractile apparatus of cardiomyocytes), and diagnostic value: a decrease in the size of the cTnT molecule as a result of fragmentation, and a possible increase in the release of these fragments into the bloodstream.

Changes in acidity (pH) can also modulate the activity of intracellular proteolytic enzymes [156,157]. So, pathological conditions that disrupt myocardial metabolism, in particular myocardial ischemia, lead to a switch from aerobic myocardial metabolism to anaerobic metabolism, and an increase in the formation of lactic acid, which will shift the pH towards acidosis. Under conditions of acidosis, then, proteolytic and proapoptotic enzymes will be activated [73,147,157,158], which, through fragmentation, will promote the formation of many small fragments (molecules) of cTns, which will increase the likelihood of their release from MCs into the bloodstream.

### 2.6. Release of cTns as a Result of Increased Membrane Permeability of MCs

The membrane permeability of MCs is an important factor that plays a role in the release of cardiac marker molecules from MCs into the bloodstream. Based on the analysis of the results of existing experimental data, two main mechanisms can be distinguished that underlie the change (increase) in the membrane permeability of MCs: (1) an increase in the membrane permeability of MCs as a result of an increase in the load on the myocardium and stretching of its walls; (2) an increase in the membrane permeability of MCs as a result of myocardial ischemia, and the activation of proteolytic enzymes that can damage the cell membrane.

The first mechanism for the release of cTns was studied by Hessel et al. [159]. In their experimental study, the authors stimulated the special glycoprotein receptors of MCs (integrins) that are sensitive to myocardial stretching. To model the myocardial stretching and activation of integrins, the researchers used the RGD (Arg–Gly–Asp) tripeptide, which is a potent integrin agonist, and is part of fibronectin and other regulatory proteins of the extracellular matrix [160]. The authors particularly note that myocardial stretching is not associated with ischemic and necrotic processes in the cardiac muscle tissue, which indicates that it was the specific mechanism of myocardial wall stretching and the activation of integrins that ensured the release of cTns from viable MCs [159].

The second mechanism for increasing membrane permeability is associated with membrane damage during the ischemia of MCs. As already described above, myocardial ischemia initiates changes in the metabolism of cardiomyocytes and the acidification (acidosis) of the intracellular space of MCs, which, in turn, will lead to the activation of proteolytic and proapoptotic enzymes. These enzymes have many targets, and in addition to the specific action (fragmentation of cTns), they can obviously catalyze the proteolysis of proteins that make up cell organelles and membranes [161,162,163]. Thus, this mechanism of troponin increase is closely interrelated with the above-described mechanism (troponin increase due to increased proteolytic degradation into small fragments). In general, the increased membrane permeability and intracellular fragmentation of cTns can be considered as two interrelated and synergistic mechanisms underlying the release of cardiac troponin molecules from MCs. The degree of activity of these mechanisms is probably related to the severity of pathological processes. For example, the short-term and/or reversible ischemia of MCs during exercise or in uncomplicated sepsis may be associated with a relatively small increase in the activity of intracellular proteolytic enzymes. In this regard, the degree of increase in the serum levels of cTns will also be relatively small and dependent only on the cytoplasmic fraction of cTns (their fragmentation into small molecular fragments) and reversible membrane damage/increased membrane permeability. In pathological conditions that cause the irreversible ischemia of MCs (for example, MI or severe/complicated sepsis), serum troponin levels increase much more significantly, and the main contribution to total serum levels of cTns will be made by the structural fraction of cTns. Both the proteins of the Tn-TPM complex and the proteins of the membranes of cardiomyocytes will be more actively fragmented (cleaved), and therefore the degree of release of cTns in these pathologies will be higher. The further prognosis of patients suffering from both cardiac and non-cardiac pathologies is also associated with the degree of increase in the serum levels of cTns, which indicates the depth and nature of damage to the cardiac muscle tissue.

### 2.7. Release of cTns as a Result of Small-Scale (Subclinical) Necrosis of Cardiomyocytes

A possible mechanism underlying the release of cTns is small-scale necrotic processes, which can be caused by both ischemia and inflammatory-toxic processes, imbalances in the neurohumoral system.

So, according to some researchers, regular heavy physical exertion, myocarditis, and stressful situations can cause subclinical damage to myocardial tissue (death of single cardiomyocytes), which can subsequently be associated with the formation of relatively small areas of fibrosis and an increased risk of sudden cardiac death [164]. So, for example, the adverse effect of serious and/or intense physical activity is confirmed by a number of studies and described clinical cases, in which sudden cardiac death was recorded in athletes [165,166,167].

Some studies registered extremely high levels of cardiac markers, including cTns in the blood serum of athletes, after serious and prolonged physical activity [168,169,170], which is also a reason for discussing possible small-scale necrotic processes. A contradictory argument is a clinical study using magnetic resonance imaging with gadolinium (contrast), which revealed no signs of necrosis and sclerosis in the cardiac muscle tissue of athletes [171]. However, the limitation of this method is its relatively lower sensitivity compared to the laboratory biomarkers of myocardial necrosis and fibrosis.

Although, during psycho-emotional stress, the level of troponin increase is relatively small (rarely exceeds the levels of the 99th percentile in the isolated effect of stress), it cannot be considered a safe process [172,173]. The constant influence of stress is considered a risk for the development of cardiovascular diseases, and may be one of the triggers of MI [174,175]. A number of molecules released during stress (for example, cortisol, catecholamines) increase myocardial oxygen demand, thereby contributing to the development of the relative ischemia of MCs.

### 2.8. Release of cTns from Non-Cardiac Cells

One of the controversial but hypothetically possible mechanisms underlying the increase in serum levels of cTns is the release of these molecules from non-cardiac cells. Several experimental and clinical studies indicate the expression of cardiac troponin molecules in skeletal muscle cells [103,176,177] and the walls of large vessels [178,179], which allows us to consider these organs as possible sources of serum levels of cTns. Thus, American biochemists (Ricchiuti and Apple), using polymerase chain reaction (PCR), revealed the expression of messenger RNA of cTnT in the skeletal muscle tissue of adults suffering from end-stage chronic renal failure (CRF) and hereditary skeletal myopathy (Duchenne muscular dystrophy). CTnI messenger RNA was not detected in the skeletal muscles of patients suffering from these pathologies and in the skeletal muscles of healthy people. In addition, no signs of cTnT expression were detected in the skeletal muscles of healthy people [103], which indicates the possible expression of one type of cardiac troponin (cTnT), only in the presence of the indicated pathologies. In another study, Messner et al. confirmed the possibility of extracardiac expression of cTnT in patients with skeletal myopathies. The researchers, using PCR, found messenger RNA of cTnT in patients with primary sarcoglycanopathy and Duchenne muscular dystrophy [177]. In some patients with skeletal myopathies, in addition to cTnT messenger RNA, the expression of cTnI messenger RNA was observed [177]. However, in these studies, the authors did not measure the serum levels of cTns in patients with myopathies and renal failure. This is an important limitation of these studies because it does not answer the question: can the expression of cTns in skeletal muscles lead to an increase in the serum levels of cTns in patients with CRF or hereditary skeletal myopathies? In addition, several other studies should be mentioned, the results of which contradict the above data on non-cardiac expression [180,181,182]. For example, Bodor et al. conducted a study, and concluded that cTns are not expressed in skeletal muscle tissue in patients with Duchenne muscular dystrophy and polymyosites [180]. Other research groups led by Hammerer-Lercher and Schmid also did not find signs of expression of cTns in skeletal muscles [181,182].

A second potential non-cardiac source of cardiac troponin release is the walls of large veins (venae cavae and pulmonary veins). Some studies report only the presence of the expression of cTns in the walls of these veins, but do not describe the possible role of these troponins in diagnostics [178,179]. Hypothetically, it can be believed that the damage or stretching of the walls of these large veins can lead to the release of cardiac troponin molecules into the bloodstream.

Thus, due to the fact that data on extracardiac expression are either insufficient or contradictory, further research is needed to validate this mechanism.

The mechanisms of cardiac troponin release described above, and their diagnostic values, are summarized in Table 3.

### 2.9. Circulation of cTns in Blood Plasma: Influencing Factors and Diagnostic Value

The second major stage of the metabolic pathway of cTns is circulation in the bloodstream. At this stage, the molecules of cTns are influenced by a number of factors (activity of proteolytic enzymes, kinases, phosphatases, the state of renal function and the reticuloendothelial system, etc.), which can affect the serum levels of cTns and, therefore, their diagnostic value. The molecules of cTns released into the bloodstream are represented by a heterogeneous fraction (a significant variety of different forms of troponin molecules): free cTns; combined complexes consisting of several free forms of cTns (for example, cTnI + TnC, cTnT + cTnI, etc.), and small fragments of cTns [65,151,152,183,184,185,186,187]. All of the above forms of troponin molecules can undergo oxidation, glycosylation, phosphorylation, and dephosphorylation processes, which lead to the formation of very diverse forms (varieties) of troponin proteins. Modifications of troponin proteins can affect such an important parameter as the half-life (half-decay) of cTns. This parameter has not only fundamental, but also highly practical importance, since with an intensification of the breakdown of troponin proteins, their concentration and the “diagnostic window” can decrease, and with a weakening of the breakdown of cTns, their serum levels and the duration of the diagnostic window can increase. The researchers estimate that the half-life of cTnT in the bloodstream is approximately 2 h, however, for many other forms, the half-life is controversial and unknown [188,189,190]. The cTnI molecule is much less stable in the bloodstream since it actively undergoes the processes of oxidation, phosphorylation, and fragmentation [191,192]. The latter, in turn, as noted above for cTnT, depends on the activity of these enzymes, the presence of concomitant pathologies that can affect the activity of these enzymes, the intake of drugs that affect the catalytic activity of proteolytic enzymes, and the functional state of the organs responsible for the elimination of molecules of cTns. For example, increasing the activity of the enzyme thrombin (which has been shown to cause the specific fragmentation of cTnT) [152] can reduce the half-life of cTnT and its concentration in the bloodstream. It is logical that taking drugs that reduce thrombin activity (for example, direct thrombin inhibitors, direct and indirect anticoagulants) can increase the half-life of cTnT and the duration of the diagnostic window. As examples of the influence of other factors on the duration of the circulation of cTns in the bloodstream, the functional state of the kidneys and the reticuloendothelial system can be named. Thus, the protein molecules of cardiac markers, including cTnT and cTnI, can be captured by the reticuloendothelial system (macrophages) and are destroyed there [71,193,194]. Based on this, the following point of view comes out: an increase in the activity of the reticuloendothelial system (for example, with hypersplenism and splenomegaly) may be accompanied by an increase in the cleavage of cTns and a decrease in the half-life; and a decrease in the activity of the reticuloendothelial system, on the contrary, will lead to a weakening of the cleavage of cTns and an increase in the half-life. Renal function is also significantly associated with cardiac troponin levels and increased blood protease activity. Thus, an increase in the activity of proteolytic enzymes can lead to the formation of a large number of small fragments of troponin molecules, which, like many low molecular weight proteins, can be filtered through the three-layer glomerular (filtration) barrier of nephrons. However, the filtration rate can change both under physiological and pathological conditions, which can have a significant effect on the rate of removal of troponin fragments. With pronounced drops in filtration rate (for example, with CRF or a decrease in blood pressure), the molecules of cTns will not be filtered (removed) from the bloodstream into the urine, but will accumulate in the blood, which will lead to an increase in the half-life of cTns and the prolongation of the diagnostic window [195,196,197].

Clear evidence that cardiac troponin molecules can pass (filter) through the glomerular filter has been presented in several recent clinical studies due to the use of highly sensitive troponin immunoassays [38,71,73]. A similar transport mechanism is probably characteristic of the filtration of cTns into the oral fluid through the blood–salivary barrier, which is also supported by several pilot studies that have established a correlation between serum and salivary troponin levels [39,40,41,72].

The search for specific mechanisms of proteolytic cleavage of cTns in the bloodstream is of great practical importance, since it will optimize laboratory diagnostics: in particular, there is a possibility of developing antibodies directed against individual fragments of cTns, or introducing inhibitors of the main proteolytic enzymes that catalyze troponin proteolysis into diagnostic test systems to reduce interference and more thoroughly interpret the test results, taking into account comorbidities that affect the activity of enzymes breaking down cTns, etc. Unfortunately, the number of fundamental studies devoted to the investigation of the processes of proteolytic cleavage of cTns in the bloodstream is extremely small. To date, only one specific mechanism is known, described in the study by Katrukha et al. [65,152]. According to the results of this study, the enzyme thrombin catalyzes the cleavage of the full-length molecule of cTnT (the molecular weight is 35 kDa) in the region of the peptide bond between amino acids 68 and 69 into two fragments, one of which is larger (the molecular weight is 29 kDa), and the second—smaller (the molecular weight is 6 kDa) [152]. As noted above, any significant effect on thrombin activity (for instance, the use of anticoagulants) can influence the fragmentation of cTnT and, accordingly, its diagnostic value.

In general, a number of research groups studying the processes of proteolytic cleavage in the bloodstream report the presence of a very large number of fragments (approximately several tens) of cardiac troponin molecules, which have different sizes (molecular weights from several kDa to 30 or more kDa), stability, half-lives in the bloodstream (from several hours to a day), and conditions of formation (physiological conditions, the degree of severity and progression of ischemia, reperfusion time, etc.) [151,152,183,184,185,186,187]. Vylegzhanina et al. studied the composition of troponin complexes in patients with MI [187]. The researchers have identified the following main forms of cTns in MI: a ternary complex consisting of full-size cTnT and cTnI and TnC; a ternary complex consisting of truncated cTnI and integral cTnT and TnC; a binary complex consisting of truncated cTnI and TnC, as well as a number of short fragments of cTnT and cTnI, formed mainly from the central part of the molecules. As MI progressed, there was a decrease in the number of ternary complexes consisting of full-size cTns, and an increase in the number of ternary and binary complexes consisting of truncated cTns, as well as an increase in the level of fragments of cTns [187]. Such changes in the heterogeneous fraction of cTns are most likely due to an increase in the activity of proteolytic enzymes, which increase with the progression of ischemia and MI, and, accordingly, cause the fragmentation (truncation) of troponin proteins.

Very interesting data are presented by the researchers Zahran et al., who studied the degree of proteolytic degradation of cTnI in patients with varying degrees of ischemia and damage to cardiac muscle tissue [186]. The researchers noted that the degree of proteolytic cleavage of cTnI increases with an increase in the severity of ischemia and myocardial injury: the highest degree of proteolytic cleavage of cTnI was characteristic of patients with ST-segment elevation MI, while in patients with non-ST-segment elevation MI, the degree of cTnI degradation was significantly lower. The authors also found a decrease in the degree of proteolytic degradation of cTnI after reperfusion, which can probably be used to assess the quality of reperfusion. It is quite remarkable that the degree of proteolytic degradation of cTnI had a higher diagnostic value in MI than the total serum concentration of cTnI [186].

Summing up the role of the stage of cTns circulation, we should once again emphasize its potentially high diagnostic value in practical medicine. At the moment, this stage is a relatively poorly studied area of the biology of cTns. The main directions of further work in this area, in my opinion, should be as follows:

(1) Study of the fundamental specific mechanisms of proteolytic degradation of cTns in the bloodstream, both under normal conditions and under the conditions of simulated concomitant pathologies. This requires a targeted and thorough study of the potential effect of individual serum proteolytic enzymes (for example, the specific thrombin-mediated degradation of cTnT).
(2) Search for specific fragments of cTns, which are released at the earliest possible time after the onset of myocardial ischemia and the creation of antibodies to them, which will increase the sensitivity and specificity of troponin immunoassays.
(3) Search for specific fragments of cTns, which have a small molecular weight and are able to pass through the glomerular and blood-salivary barriers. The creation of antibodies to these fragments will make it possible to develop specific highly sensitive test systems for the analysis of non-invasive biological fluids (urine and oral fluid) and for the introduction of new methods of non-invasive diagnostics and monitoring of cardiovascular pathologies, including MI, into routine clinical practice.
(4) Study and identification of potentially possible specific mechanisms of proteolytic cleavage of cTns under the action of other (non-ischemic) factors. This will allow the development of specific troponin immunoassays to identify those fragments that, for example, will increase exclusively with the stretching of the myocardium or exclusively with an increase in the activity of the adrenergic nervous system and an increase in β-AR stimulation, etc. Thus, it will be possible to carry out a more specific diagnosis of non-ischemic myocardial damage in some physiological and pathological conditions not associated with ischemia of the cardiac muscle tissue.

### 2.10. Removal of cTns from the Bloodstream: Mechanisms and Diagnostic Value

The final stage of the metabolic pathway of cTns in the bloodstream is as important as the other two stages (release and circulation). Both of these stages are closely related to the terminal stage of the metabolic pathway of cTns. So, for example, when small fragments are released from cardiomyocytes (as a result of the intracellular proteolytic cleavage of cTns), they will obviously be almost immediately removed from the bloodstream by filtration through the glomerular and blood–salivary barriers. When larger fragments of cTns and/or binary and ternary complexes are released, the filtration of these molecules is unlikely due to their large size (molecular weight).

The circulation of cTns is equally closely related to the removal of these molecules. So, for example, with a higher activity of serum proteolytic enzymes, the process of degradation of cardiac troponin molecules will be more active, which will lead to the more rapid formation of small troponin molecules and their filtration (removal) from the bloodstream.

In general, today, the mechanism of filtering cTns through the glomerular barrier is one of the main and definitively proven ways to remove cTns from the bloodstream. The inferential (indirect) evidence is that when the filtration rate decreases (for example, in CRF), cardiac troponin molecules accumulate in the bloodstream and their serum levels begin to rise sharply in those patients who do not have any signs of cardiovascular pathology and damage of MCs. The more the renal function is suppressed (i.e., the lower the filtration rate is), the higher the concentration of cTns in the bloodstream rises [196,197,198]. In other pathological conditions that are accompanied by the inhibition of the filtration rate, for example, in sepsis, serum levels of cTns are positively correlated with serum creatinine levels [199,200], which also accumulate due to a decrease in the filtration capacity of nephrons. From a pathogenetic point of view, any conditions accompanied by a drop in the filtration rate can contribute to the accumulation of cTns. This fact, of course, should be taken into account by medical practitioners when interpreting the results.

Recent clinical studies by several research groups can be considered as valuable evidence of the existence of a mechanism for the elimination of cTns across the filtration barrier [38,71]. A key feature of these studies is the use of highly sensitive troponin immunoassays, which can detect small concentrations (from several ng/L to several tens of ng/L) of cTns in urine. According to these studies, it is also noteworthy that there is a possibility for the non-invasive assessment of myocardial damage in arterial hypertension and diabetes mellitus [38,71], which is very convenient for non-hospital and outpatient settings. This will allow one to monitor the patient’s condition, assess the prognosis, and, on its basis, choose/correct the tactics of the further management of patients, including their treatment. However, it should be noted that these methods have not yet been completely validated and research work in this direction should be continued before introducing new non-invasive methods for diagnosing and monitoring cardiovascular pathologies in routine clinical practice.

One of the key and very labile factors affecting the glomerular filtration rate (including the rate of removal of cTns from the bloodstream) is blood pressure. So, with a decrease in blood pressure, the filtration rate will slow down and the degree of removal of cTns from the bloodstream will decrease. This mechanism, in particular, can contribute to the fact that the molecules of cTns will increase much more and circulate in the bloodstream for longer in pathological conditions, accompanied by a sharp drop in blood pressure. This can be typical for large focal myocardial infarctions, which are often accompanied by a sharp decrease in blood pressure (cardiogenic shock), and the degree of increase/duration of circulation of cTns in the bloodstream can be considered as a prognostically unfavorable sign [201]. With an increase in blood pressure, the filtration rate may increase, and more cardiac troponin molecules will be filtered from the bloodstream into the urine. The evidence for a possible role of this mechanism comes from a clinical study showing that urinary cTn levels are higher in hypertensive patients than in those with normal blood pressure, or those taking antihypertensive drugs [71].

Another method of cTns removal is associated with the activity of the reticuloendothelial system, the cells of which capture the protein molecules of cardiac markers from the bloodstream, and cause their intracellular proteolytic cleavage [193,194,202,203]. The clinical significance of this mechanism for removing cTns (as opposed to the mechanism for removing cTns through the glomerular filter) is difficult to judge, since there are no similar well-controlled clinical studies confirming the possibility of a significant increase in serum levels of cTns in the case of reticuloendothelial system dysfunction. In addition, in contrast to renal failure, dysfunctions of the components of the reticuloendothelial system are much less common.

As noted earlier, the proteolytic cleavage of cTn molecules in the bloodstream is an extremely understudied mechanism for the elimination of cTns. As a result of this mechanism, a large number of small fragments of cTns are formed, which can be immunoreactive (can interact with antibodies and be detected by immunoassays) and non-immunoreactive fragments (which will not interact with antibodies). From the point of view of laboratory diagnostics, non-immunoreactive fragments of cTns can be considered to be already removed from the bloodstream, since they will not bind to antibodies, and thus will not have an effect on the result of the laboratory diagnostics of MI or any other pathology. To elucidate the mechanism of cTns removal by proteases, well-controlled basic research is needed to thoroughly investigate the role of individual serum proteolytic enzymes in the degradation of cTn molecules in the bloodstream.

Thus, three main mechanisms of elimination of cTns from the bloodstream can be distinguished: (1) the elimination (filtration) of cTns through the glomerular barrier, (2) the removal of cTns from the bloodstream by cells of the reticuloendothelial system, (3) the proteolytic cleavage of cTns molecules and/or their fragments in the bloodstream to non-immunoreactive forms. Taking into account the analysis of the literature, the main mechanism for the removal of cTns, in my opinion, is the elimination of cTns through a three-layer filtration (glomerular) barrier. This mechanism can have a significant impact on the diagnostics of cardiovascular diseases, including MI, since the impaired removal of cTns from the bloodstream is often accompanied by a significant increase in the serum levels of cTns. In addition, many patients have comorbid pathologies, among which kidney damage (chronic renal failure) is relatively common. Many other common diseases, for example, diabetes mellitus and sepsis, are also often complicated by renal failure. Thus, they may increase the serum levels of cTns in patients with no signs of cardiovascular diseases.

The removal of cTns from the bloodstream by glomerular filtration may have an important impact on rapid algorithms for the diagnostics/exclusion of MI. Thus, the research group led by Kavsak reported that the currently established upper threshold levels of cTns (99th percentile) for the diagnostics/exclusion of MI can be used only for patients with an optimal glomerular filtration rate (≥90 mL/min) [204]. In patients who have lower glomerular filtration rate values, cTns levels will increase due to impaired elimination, which can lead to the overdiagnosis of MI if medical practitioners do not take renal function (filtration rate value) into account. Thus, it is necessary to stratify the threshold values of cTns, taking into account different values of the filtration rate and, in particular, to develop special algorithms for the diagnostics/exclusion of MI for patients who suffer from concomitant CRF.

Finally, the filtration of troponin fragments through the blood–brain barrier into the cerebrospinal fluid and through the blood–salivary barrier into saliva can be considered as additional potential mechanisms for the removal of cTns. As evidence of the existence of these mechanisms, one can consider studies [39,40,41,72,78,79,80] which reported on the detection of cTns in the cerebrospinal fluid and saliva. The investigation of cTns in the cerebrospinal fluid can be used in forensic medicine, as well as the investigation of cTns in saliva—in clinical practice for diagnostics and monitoring of cardiovascular pathologies, including MI. Overall, more research is needed to validate these diagnostic capabilities.

### 2.11. Circadian Rhythms of cTns: Possible Mechanisms of Formation and Diagnostic Role

The activity of many systems (organs, tissues, and cells) of our body changes cyclically during the day (with the change of day and night), commonly called circadian or diurnal rhythms. Circadian rhythms are an evolutionarily developed mechanism necessary to maintain the optimal functioning of the body and adapt to changing environmental conditions [205].

Due to the fact that the tissues and cells of our body change, there is a change in the concentration of a number of molecules (for example, hormones, metabolic products), which are produced or metabolized by these tissues and cells. Many of these molecules are laboratory biomarkers, the concentration of which is used to diagnose diseases [206,207]. This must be taken into account in routine clinical practice, since changes in the concentration of biomarkers caused by natural circadian rhythms can be mistakenly interpreted as diagnostic signs and, accordingly, lead to diagnostic errors. Certain hormones, the release of which vary from day to night, can affect a number of other laboratory parameters that must also be considered when interpreting laboratory diagnostic results.

Recent clinical studies have reported that the bloodstream levels of cTns are dependent on circadian rhythms. These studies used highly sensitive troponin immunoassays able to detect small fluctuations in the concentration of cTns in the bloodstream (at the level of several ng/L) [50,51,208,209,210]. For example, Klinkenberg et al. revealed changes in cTnT concentration (detected by a highly sensitive method) in patients without signs of cardiovascular diseases. At the same time, the maximum levels of cTns were recorded in the morning (16.2 ng/L at 8:30), and the minimum—in the evening (12.1 ng/L at 19:30). In addition, when analyzing the hourly curve of the serum levels of cTns, the researchers found very regular and gradual changes: for example, from the maximum morning concentrations of cTns, there was a gradual decrease to the evening (minimum) concentrations of cTns, and then there was a gradual increase in concentrations to the maximum morning values [208]. However, the researchers noted that such relatively minor circadian fluctuations in cTns would not have a significant impact on diagnostic algorithms for MI, but should be considered for screening purposes. The levels of cTnI (also detected by a highly sensitive immunoassay) changed over a 24-h period by no more than 1 ng/L, i.e., had no significant circadian rhythms [208]. However, this study investigated the circadian rhythms of cTns only in healthy people, but in conditions of concomitant pathologies (especially with damage to those organs that affect the metabolism of cTns), fluctuations in the circadian rhythms of cTns can be much more pronounced. Thus, in patients with concomitant CRF, cTnT and cTnI concentrations changed more significantly during the day. Der Linden et al. [209] reported that the maximum fluctuations in cTnT concentration in a patient with CRF during the 24-h investigation period were about 50 ng/L, while the fluctuations in cTnT levels for one hour were about 20 ng/L, which, by the way, is a very significant contribution to the laboratory diagnosis of MI. So, for example, if we take into account modern algorithms for the diagnostics of non-ST-segment elevation MI (Table 1) [33] (where the change in the levels of cTns within 1–2 h by only 5–10 ng/L is diagnostically significant), we can say that that cTnT circadian rhythms may affect the diagnostics of MI and contribute to overdiagnosis [209]. CTnI levels showed slightly higher fluctuations in concentration during the day compared to the study by Klinkenberg et al. [208], however, they would not reach the thresholds of 5–10 ng/L, and thus would not have a significant effect on the one- and two-hour algorithms of MI diagnostics.

The precise mechanisms of the formation of circadian rhythms of cTns are unknown, however, it can be assumed that they will be associated with changes in the functional activity of those organs, tissues, and cells that can somehow affect the metabolic pathway of cTns, in particular, the stages of their release into the bloodstream, the stage of circulation (for example, the effect on the activity of proteolytic enzymes that cause the cleavage of cTns in the bloodstream), or the elimination stage (for example, the effect on the functional state of the kidneys). Among the most probable mechanisms for the formation of circadian rhythms of cTns, in my opinion, there are circadian fluctuations in the activity of the cortex and medulla of the adrenal glands, of the thyroid gland, and the activity of enzymes of the hemostatic system [58,211,212,213,214,215,216,217,218]. A possible rationale for the formation of circadian rhythms is that peak troponin concentrations occur in the morning period, being the period of the maximum activity of the adrenal glands (producing elevated levels of catecholamines, cortisol), the thyroid gland (producing thyroid hormones, which can enhance the effects of catecholamines on MCs). The increased activity of these organs also coincides with their main effects on the cardiovascular system, namely, in the morning period, patients have the highest heart rate, and the blood pressure is higher than in the evening–night period. In general, the increased activity of these organs is a kind of adaptive and evolutionary developed mechanism, which is necessary to maintain the period of wakefulness. However, we should take into account the negative impact of these organs and their metabolic products (for example, catecholamines, cortisol) on MCs. The evidence of the adverse effects of cortisol on MCs is a clinical study that demonstrates that increased levels of the stress hormone (cortisol) are associated with increased levels of cTnT [172]. In addition, a number of researchers associate the increased high activity of the sympathoadrenal system with a larger size of the focus of myocardial necrosis in MI, the incidence of acute cardiovascular diseases, and an unfavorable prognosis [219,220,221,222,223,224,225,226].

A possible explanation of the reason for the fact that the circadian rhythms of cTnT are more significant than the circadian rhythms of cTnI is their biochemical features, in particular, the fact that the volume of the cytoplasmic fraction of cTnT is almost twice the volume of the cytoplasmic fraction of cTnI (approximately 7–8% versus 3–4%) [57,58]. Thus, the cytoplasmic fraction of cTnT is more “mobile”, and can be released into the bloodstream with an increase in the effect of a number of factors on the myocardium. There is a need for further clinical studies validating the circadian rhythms of cTns and their effect on the diagnostics of cardiovascular diseases, including MI, and for fundamental research clarifying the molecular mechanisms of the formation of circadian rhythms of cTns.

## 3. Conclusions and Future Perspectives

The metabolic pathway of cTns includes three main stages (release of cTns from MCs, circulation of cTns in blood plasma, removal of cTns from the bloodstream), each of which can have a very significant effect on the serum levels of cTns, i.e., on their diagnostic value. The format of the review, starting with the release of troponin in blood, and concluding with the metabolism/filtration of troponins, provides a comprehensive yet logically easy way for the reader to approach our current knowledge in the framework of understanding the basic mechanisms by which troponins are produced and processed.

Based on the analysis of the current literature, the important role of biology and all the stages of metabolism (release, circulation, removal) of cTns in laboratory diagnostics should be noted. It is necessary to continue studying the biology and metabolism of cTns, because this will improve the differential diagnosis of MI, and create a new application of cTns immunoassays in current clinical practice.

It should be noted that many new views on the biology, metabolism, and diagnostic value of cTns (in particular, the role of circadian rhythms, gender- and age-related characteristics of concentrations, the possibility of detecting cTns in urine and saliva, etc.) were formed as a result of an increase in the sensitivity of troponin immunoassays.

Unfortunately, today, many stages of the metabolic pathway of cTns and factors influencing the metabolic pathway of cTns are extremely poorly understood, and are hypothetical and/or contradictory. In particular, the specific mechanisms of the release of cTns from the myocardium into the bloodstream, as affected by physiological conditions and characteristics (physical exertion, stress, circadian fluctuations in the activity of organs, and tissues that influence the release of cTns molecules) and non-ischemic pathologies, which are often accompanied by an increase in the concentration of cTns in the bloodstream, are not very well known. Moreover, factors affecting the circulation of cTns molecules in the bloodstream, in particular, enzymes involved in the metabolism (fragmentation) of troponin molecules, remain unknown. The mechanisms of filtration (transport) of cardiac troponin molecules from the bloodstream to other biological fluids are not investigated, and, accordingly, these possibilities of non-invasive diagnostics have not been validated. Thus, the study of the biology and metabolism of cTns and potential factors influencing it is a relatively large and poorly studied area for further research, which is needed to optimize diagnostics and validate new diagnostic capabilities.

## Figures and Tables

**Table 1:** Current diagnostic algorithms for confirmation/exclusion of NSTEMI (0 → 1 h and 0 → 2 h), approved by the ESC.

**One-Hour NSTEMI Diagnostic Algorithm**					
**Troponin Immunoassay**, **Company (Manufacturer)**	**Biomarker Concentration That Indicates an Extremely Low Probability of an NSTEMI Diagnosis**, **ng/L**	**Biomarker Concentration That Indicates a Low Probability of an NSTEMI Diagnosis**, **ng/L**	**Changes in Biomarker Concentration after 1 h at which a Diagnosis of NSTEMI Should be Excluded**, **ng/L**	**Biomarker Concentration That Indicates a High Probability of an NSTEMI Diagnosis**, **ng/L**	**Changes in Biomarker Concentration after 1 h at which a Diagnosis of NSTEMI Should be Confirmed**, **ng/L**
**hs-cTnT (Elecsys; Roche)**	<5	<12	<3	≥52	≥5
**hs-cTnI (Architect; Abbott)**	<4	<5	<2	≥64	≥6
**hs-cTnI (Centaur; Siemens)**	<3	<6	<3	≥120	≥12
**hs-cTnI (Access; Beckman Coulter)**	<4	<5	<4	≥50	≥15
**hs-cTn I (Clarity; Singulex)**	<1	<2	<1	≥30	≥6
**hs-cTn I (Vitros; Clinical Diagnostics)**	<1	<2	<1	≥40	≥4
**hs-cTnI (Pathfast; LSI Medience)**	<3	<4	<3	≥90	≥20
**Two-Hour NSTEMI Diagnostic Algorithm**					
**Troponin immunoassay, company (manufacturer)**	**Biomarker concentration that indicates an extremely low probability of an NSTEMI diagnosis**, **ng/L**	**Biomarker concentration that indicates a low probability of an NSTEMI diagnosis**, **ng/L**	**Changes in biomarker concentration after 2 h at which a diagnosis of NSTEMI should be excluded**, **ng/L**	**Biomarker concentration that indicates a high probability of an NSTEMI diagnosis**, **ng/L**	**Changes in biomarker concentration after 2 h at which a diagnosis of NSTEMI should be confirmed**, **ng/L**
**hs-cTnT (Elecsys; Roche)**	<5	<14	<4	≥52	≥10
**hs-cTnI (Architect; Abbott)**	<4	<6	<2	≥64	≥15
**hs-cTnI (Centaur; Siemens)**	<3	<8	<7	≥120	≥20
**hs-cTnI (Access; Beckman Coulter)**	<4	<5	<5	≥50	≥20
**hs-cTn I (Clarity; Singulex)**	<1	to be determined	to be determined	≥30	to be determined
**hs-cTn I (Vitros; Clinical Diagnostics)**	<1	to be determined	to be determined	≥40	to be determined
**hs-cTn I (Pathfast; LSI Medience)**	<3	to be determined	to be determined	≥90	to be determined

**Table 2:** Biological fluids in which the molecules of cTns are detected and the diagnostic value.

Human Biological Fluids	Diagnostic Role	References
**Blood (whole, serum, plasma)**	It is the main biological fluid used to diagnose MI and assess the prognosis of patients suffering from non-ischemic cardiac (myocardites, Takotsubo syndrome, cardiomyopathies, etc.) and non-cardiac (sepsis, renal failure, neurogenic pathologies, etc.) pathologies that cause damage to MCs.	[15,16,17,23,24,25,26]
**Urine**	Molecules of cTns can be detected in this biological fluid via highly sensitive test systems. Increased cTns levels have a high prognostic value in diabetes mellitus and arterial hypertension. The method of obtaining this biological fluid is non-invasive, which has a number of advantages over the use of blood. It should be noted that the possibilities of examination of highly sensitive cTns in urine are still poorly studied and have not been finally validated. Further research is needed before the introduction of this method into clinical practice.	[38,71]
**Oral fluid**	The levels of cTns in oral fluid increase in MI and moderately correlate with serum troponin levels; therefore, further study of this area of non-invasive diagnostics is very promising.	[39,40,41,70,72]
**Pericardial fluid and cerebrospinal fluid**	Molecules of cTns are detected in pericardial fluid and cerebrospinal fluid via moderately sensitive and highly sensitive test systems and, according to some studies, may correlate with serum levels of cTns. Increased troponin levels in these biological fluids may reflect the degree of myocardial damage and may be used in forensic medicine to determine the cause of death. Thus, according to Hernández-Romero et al., the concentration of troponin I in the pericardial fluid and the ratio of pericardial and serum levels of troponin I are associated with the cause of death. Highest cTnI ratio values were shown for AMI deaths, followed by asphyctic, traumatic and deaths by other natural causes [76]. However, due to the relative paucity of such studies, further investigation of these possibilities is necessary.	[76,77,78,79,80,81,82]
**Amniotic fluid**	cTns molecules can be detected in amniotic fluid via moderately sensitive and highly sensitive immunoassays. Increased cTns levels may indicate chronic fetal hypoxia, abnormal development of the cardiovascular system and fetal myocardial injury, and an increased risk of fetal death during the intrauterine growth period. However, it is worth noting that such studies are few in number. Further research is needed to clarify the diagnostic capabilities of amniotic fluid.	[83,84,85,86]

**Table 3:** Release of cTns from MCs: mechanisms and diagnostic value.

Mechanism	Diagnostic Value	References
**Myocardial cell necrosis**	This is the main proven mechanism underlying the increase in cTns in MI. Cardiomyocyte necrosis will result in the release of all molecules (biomarkers) from the cell into the bloodstream.	[14,15,16]
**Release of cTns as a result of the processes of regeneration and renewal of MCs**	The renewal of MCs gradually occurring throughout life, hypothetically, may be associated with normal (less than the upper limit of the 99th percentile) concentrations of cTns in the bloodstream.	[105,106,107,108,109,110,111,112,113,114]
**Release of cTns as a result of apoptosis of MCs**	It has been proven that apoptosis of cardiomyocytes (without signs of necrosis) is accompanied by an increase in the serum concentration of cTns. Thus, any physiological (physical activity, old age) and pathological (heart failure, arterial hypertension, chronic obstructive pulmonary disease, etc.) conditions that enhance apoptosis may be accompanied by the release of cTns from cardiomyocytes and an increase in serum levels.	[117,118,119,120,127,128,129,130,131,132,133,134,135,136]
**Release of cTns as a result of the formation of membrane vesicles on the surface of MCs**	Membrane vesicles (blebbing vesicles) formed on the surface of the plasma membrane of cardiomyocytes, hypothetically, may contain cytoplasmic proteins, including cTns. The number of membrane vesicles increases during ischemia of MCs and may be associated with the release of cTns into the bloodstream.	[137,138,139,140,141,142]
**Intracellular proteolytic degradation of cTns molecules into small fragments and the release of the latter through the intact membrane of MCs**	Molecules of cTns can be fragmented/destroyed by the action of certain proteolytic enzymes: calpain, thrombin, matrix metalloproteinases. As a result of the action of these enzymes, there can form small fragments of troponin molecules, which, due to their size, have a higher probability of release from the cell. This mechanism may have high clinical significance: for example, all those physiological and pathological conditions and/or drugs that affect the activity of these proteolytic enzymes can also affect the release of cTns and their concentration in the bloodstream.	[147,148,149,150,151,152,153,154,155]
**Release of cTns as a result of increased membrane permeability of MCs**	An increase in the release of cTns molecules into the bloodstream is observed in case of an increase in the membrane permeability of MCs, which is characteristic of myocardial ischemia, an increase in preload and stretching of the heart wall.	[159,160,161,162,163]
**Release of cTns as a result of small-scale (subclinical) necrosis of cardiomyocytes**	The death of a small number of cardiomyocytes may not manifest itself clinically and instrumentally (since these are relatively low-sensitivity methods), but highly sensitive methods of detection can register such subclinical lesions. Possible causes of subclinical necrosis of cardiomyocytes are ischemia, inflammatory-toxic processes and imbalances in the neuroendocrine system.	[164,165,166,167,168,169,170,171,172,173,174,175]
**Release of cTns from non-cardiac cells**	This is a controversial mechanism of increased levels of cTns in the bloodstream. In the literature, there are works confirming the expression of cTns in skeletal muscle tissue in patients with CRF and hereditary skeletal myopathies, as well as studies that refute this hypothesis.	[103,104,176,177,178,179,180,181,182]

cTns—cardiac troponins, MCs—myocardial cells.

## Data Availability

Not applicable.

## References

[1] Takeda S. (2005). Crystal structure of troponin and the molecular mechanism of muscle regulation. J. Electron. Microsc. Tokyo.

[2] Chaulin A.M. (2022). Main analytical characteristics of laboratory methods for the determination of cardiac troponins: A review from the historical and modern points of view. Orv. Hetil..

[3] Katrukha I.A. (2013). Human cardiac troponin complex. Structure and functions. Biochem. Mosc..

[4] Henderson C.A., Gomez C.G., Novak S.M., Mi-Mi L., Gregorio C.C. (2017). Overview of the Muscle Cytoskeleton. Compr. Physiol..

[5] Chaulin A. (2021). Clinical and Diagnostic Value of Highly Sensitive Cardiac Troponins in Arterial Hypertension. Vasc. Health Risk Manag..

[6] Chaulin A.M. (2021). Cardiac troponins: Current information on the main analytical characteristics of determination methods and new diagnostic possibilities. Medwave.

[7] Wei B., Jin J.P. (2011). Troponin T isoforms and posttranscriptional modifications: Evolution, regulation and function. Arch. Biochem. Biophys..

[8] Jin J.P. (2016). Evolution, Regulation, and Function of N-terminal Variable Region of Troponin T: Modulation of Muscle Contractility and Beyond. Int. Rev. Cell Mol. Biol..

[9] Chaulin A. (2021). Cardiac Troponins: Contemporary Biological Data and New Methods of Determination. Vasc. Health Risk Manag..

[10] Wang X.Y., Zhang F., Zhang C., Zheng L.R., Yang J. (2020). The Biomarkers for Acute Myocardial Infarction and Heart Failure. Biomed. Res. Int..

[11] Smith J.N., Negrelli J.M., Manek M.B., Hawes E.M., Viera A.J. (2015). Diagnosis and management of acute coronary syndrome: An evidence-based update. J. Am. Board Fam. Med..

[12] Henderson R.A. (2013). Acute coronary syndrome: Optimising management through risk assessment. Clin. Med..

[13] Chaulin A.M., Grigorieva Y.u.V., Pavlova T.V., Duplyakov D.V. (2020). Diagnostic significance of complete blood count in cardiovascular patients; Samara State Medical University. Russ. J. Cardiol..

[14] Makki N., Brennan T.M., Girotra S. (2015). Acute coronary syndrome. J. Intensive Care Med..

[15] Thygesen K., Alpert J.S., Jaffe A.S., Simoons M.L., Chaitman B.R., White H.D., Thygesen K., Alpert J.S., White H.D., Writing Group on the Joint ESC/ACCF/AHA/WHF Task Force for the Universal Definition of Myocardial Infarction (2012). Third universal definition of myocardial infarction. J. Am. Coll. Cardiol..

[16] Thygesen K., Alpert J.S., Jaffe A.S., Chaitman B.R., Bax J.J., Morrow D.A., White H.D., Executive Group on behalf of the Joint European Society of Cardiology (ESC)/American College of Cardiology (ACC)/American Heart Association (AHA)/World Heart Federation (WHF) Task Force for the Universal Definition of Myocardial Infarction (2018). Fourth Universal Definition of Myocardial Infarction (2018). Circulation.

[17] Chaulin A.M., Duplyakov D.V. (2020). MicroRNAs in atrial fibrillation: Pathophysiological aspects and potential biomarkers. Int. J. Biomed..

[18] Eckner D., Pauschinger M., Ademaj F., Martinovic K. (2020). Klinische Bedeutung der 4. Universellen Definition des Myokardinfarkts [Clinical implications of the fourth universal definition of myocardial infarction]. Herz.

[19] Apple F.S., Sandoval Y., Jaffe A.S., Ordonez-Llanos J., IFCC Task Force on Clinical Applications of Cardiac Bio-Markers (2017). Cardiac Troponin Assays: Guide to Understanding Analytical Characteristics and Their Impact on Clinical Care. Clin. Chem..

[20] Aakre K.M., Saeed N., Wu A.H.B., Kavsak P.A. (2020). Analytical performance of cardiac troponin assays—Current status and future needs. Clin. Chim. Acta.

[21] Chaulin A.M., Duplyakov D.V. (2020). Arrhythmogenic effects of doxorubicin. Complex Issues Cardiovasc. Dis..

[22] Chaulin A.M., Abashina O.E., Duplyakov D.V. (2020). Pathophysiological mechanisms of cardiotoxicity in chemotherapeutic agents. Russ. Open Med. J..

[23] Chuang A.M., Nguyen M.T., Kung W.M., Lehman S., Chew D.P. (2020). High-sensitivity troponin in chronic kidney disease: Considerations in myocardial infarction and beyond. Rev. Cardiovasc. Med..

[24] Chaulin A.M., Duplyakov D.V. (2020). Increased natriuretic peptides not associated with heart failure. Russ. J. Cardiol..

[25] Stavroulakis G.A., George K.P. (2020). Exercise-induced release of troponin. Clin. Cardiol..

[26] Chaulin A.M. (2021). Elevation Mechanisms and Diagnostic Consideration of Cardiac Troponins under Conditions Not Associated with Myocardial Infarction. Part 1. Life.

[27] Chaulin A.M. (2021). Elevation Mechanisms and Diagnostic Consideration of Cardiac Troponins under Conditions Not Associated with Myocardial Infarction. Part 2. Life.

[28] Chaulin A.M. (2022). False-Positive Causes in Serum Cardiac Troponin Levels. J. Clin. Med. Res..

[29] Lindner G., Pfortmueller C.A., Braun C.T., Exadaktylos A.K. (2014). Non-acute myocardial infarction-related causes of elevated high-sensitive troponin T in the emergency room: A cross-sectional analysis. Intern. Emerg. Med..

[30] Wu W., Li D.X., Wang Q., Xu Y., Cui Y.J. (2018). Relationship between high-sensitivity cardiac troponin T and the prognosis of elderly inpatients with non-acute coronary syndromes. Clin. Interv. Aging.

[31] Askin L., Tanriverdi O., Turkmen S. (2020). Clinical importance of high- sensitivity troponin T in patients without coronary artery disease. North. Clin. Istanb..

[32] Qin Z.J., Wu Q.Y., Deng Y., Li X., Wei X.D., Tang C.J., Jia J.F. (2021). Association Between High-Sensitivity Troponin T on Admission and Organ Dysfunction During Hospitalization in Patients Aged 80 Years and Older with Hip Fracture: A Single-Centered Prospective Cohort Study. Clin. Interv. Aging.

[33] Collet J.P., Thiele H., Barbato E., Barthélémy O., Bauersachs J., Bhatt D.L., Dendale P., Dorobantu M., Edvardsen T., Folliguet T. (2021). 2020 ESC Guidelines for the management of acute coronary syndromes in patients presenting without persistent ST-segment elevation. Eur. Heart J..

[34] Odsæter I.H., Grenne B., Hov G.G., Laugsand L.E., Wiseth R., Mikkelsen G. (2020). Establishing the 99th percentile of a novel assay for high-sensitivity troponin I in a healthy blood donor population. Clin. Chem. Lab. Med..

[35] Bahadur K., Ijaz A., Salahuddin M., Alam A. (2020). Determination of high sensitive cardiac troponin I 99th percentile upper reference limits in a healthy Pakistani population. Pak. J. Med. Sci..

[36] Koerbin G., Tate J., Potter J.M., Cavanaugh J., Glasgow N., Hickman P.E. (2012). Characterisation of a highly sensitive troponin I assay and its application to a cardio-healthy population. Clin. Chem. Lab. Med..

[37] Abe N., Tomita K., Teshima M., Kuwabara M., Sugawa S., Hinata N., Matsuura M., Fujiwara M., Takaya K., Hiyoshi T. (2018). Distribution of cardiac troponin I in the Japanese general population and factors influencing its concentrations. J. Clin. Lab. Anal..

[38] Chen J.Y., Lee S.Y., Li Y.H., Lin C.Y., Shieh M.D., Ciou D.S. (2020). Urine High-Sensitivity Troponin I Predict Incident Cardiovascular Events in Patients with Diabetes Mellitus. J. Clin. Med..

[39] Chaulin A.M., Karslyan L.S., Bazyuk E.V., Nurbaltaeva D.A., Duplyakov D.V. (2019). Clinical and Diagnostic Value of Cardiac Markers in Human Biological Fluids. Kardiologiia.

[40] Chaulin A.M., Duplyakova P.D., Bikbaeva G.R., Tukhbatova A.A., Grigorieva E.V., Duplyakov D.V. (2020). Concentration of high-sensitivity cardiac troponin I in the oral fluid in patients with acute myocardial infarction: A pilot study. Russ. J. Cardiol..

[41] Mirzaii-Dizgah I., Riahi E. (2013). Salivary high-sensitivity cardiac troponin T levels in patients with acute myocardial infarction. Oral Dis..

[42] Garcia-Osuna A., Gaze D., Grau-Agramunt M., Morris T., Telha C., Bartolome A., Bishop J.J., Monsalve L., Livingston R., Estis J. (2018). Ultrasensitive quantification of cardiac troponin I by a Single Molecule Counting method: Analytical validation and biological features. Clin. Chim. Acta.

[43] Giannitsis E., Mueller-Hennessen M., Zeller T., Schuebler A., Aurich M., Biener M., Vafaie M., Stoyanov K.M., Ochs M., Riffel J. (2020). Gender-specific reference values for high-sensitivity cardiac troponin T and I in well-phenotyped healthy individuals and validity of high-sensitivity assay designation. Clin. Biochem..

[44] Rocco E., La Rosa G., Liuzzo G., Biasucci L.M. (2019). High-sensitivity cardiac troponin assays and acute coronary syndrome: A matter of sex?. J. Cardiovasc. Med..

[45] Romiti G.F., Cangemi R., Toriello F., Ruscio E., Sciomer S., Moscucci F., Vincenti M., Crescioli C., Proietti M., Basili S. (2019). Sex-Specific Cut-Offs for High-Sensitivity Cardiac Troponin: Is Less More?. Cardiovasc. Ther..

[46] Monneret D., Gellerstedt M., Bonnefont-Rousselot D. (2018). Determination of age- and sex-specific 99th percentiles for high-sensitive troponin T from patients: An analytical imprecision- and partitioning-based approach. Clin. Chem. Lab. Med..

[47] Bohn M.K., Higgins V., Kavsak P., Hoffman B., Adeli K. (2019). High-Sensitivity Generation 5 Cardiac Troponin T Sex- and Age-Specific 99th Percentiles in the CALIPER Cohort of Healthy Children and Adolescents. Clin. Chem..

[48] Boeddinghaus J., Nestelberger T., Twerenbold R., Neumann J.T., Lindahl B., Giannitsis E., Sörensen N.A., Badertscher P., Jann J.E., Wussler D. (2018). Impact of age on the performance of the ESC 0/1h-algorithms for early diagnosis of myocardial infarction. Eur. Heart J..

[49] Gore M.O., Seliger S.L., Defilippi C.R., Nambi V., Christenson R.H., Hashim I.A., Hoogeveen R.C., Ayers C.R., Sun W., McGuire D.K. (2014). Age- and sex-dependent upper reference limits for the high-sensitivity cardiac troponin T assay. J. Am. Coll. Cardiol..

[50] Fournier S., Iten L., Marques-Vidal P., Boulat O., Bardy D., Beggah A., Calderara R., Morawiec B., Lauriers N., Monney P. (2017). Circadian rhythm of blood cardiac troponin T concentration. Clin. Res. Cardiol..

[51] Klinkenberg L.J., van Dijk J.W., Tan F.E., van Loon L.J., van Dieijen-Visser M.P., Meex S.J. (2014). Circulating cardiac troponin T exhibits a diurnal rhythm. J. Am. Coll. Cardiol..

[52] Chaulin A.M., Duplyakov D.V. (2021). High-sensitivity cardiac troponins: Circadian rhythms. Cardiovasc. Ther. Prev..

[53] Chaulin A.M., Abashina O.E., Duplyakov D.V. (2021). High-sensitivity cardiac troponins: Detection and central analytical characteristics. Cardiovasc. Ther. Prev..

[54] Eggers K.M., Lindahl B. (2017). Impact of Sex on Cardiac Troponin Concentrations-A Critical Appraisal. Clin. Chem..

[55] Sedighi S.M., Prud’Homme P., Ghachem A., Lepage S., Nguyen M., Fulop T., Khalil A. (2019). Increased level of high-sensitivity cardiac Troponin T in a geriatric population is determined by comorbidities compared to age. Int. J. Cardiol. Heart Vasc..

[56] Hickman P.E., Abhayaratna W.P., Potter J.M., Koerbin G. (2019). Age-related differences in hs-cTnI concentration in healthy adults. Clin. Biochem..

[57] Chaulin A.M., Duplyakova P.D., Duplyakov D.V. (2020). Circadian rhythms of cardiac troponins: Mechanisms and clinical significance. Russ. J. Cardiol..

[58] Chaulin A.M., Duplyakov D.V. (2021). On the potential effect of circadian rhythms of cardiac troponins on the diagnosis of acute myocardial infarction. Signa Vitae..

[59] Vogiatzis I. (2018). Circadian rhythm of cardiac troponins. Does it really exist?. Int. J. Cardiol..

[60] Zaninotto M., Padoan A., Mion M.M., Marinova M., Plebani M. (2020). Short-term biological variation and diurnal rhythm of cardiac troponin I (Access hs-TnI) in healthy subjects. Clin. Chim. Acta.

[61] Wildi K., Singeisen H., Twerenbold R., Badertscher P., Wussler D., Klinkenberg L.J.J., Meex S.J.R., Nestelberger T., Boeddinghaus J., Miró Ò. (2018). Circadian rhythm of cardiac troponin I and its clinical impact on the diagnostic accuracy for acute myocardial infarction. Int. J. Cardiol..

[62] Tate J.R., Bunk D.M., Christenson R.H., Katrukha A., Noble J.E., Porter R.A., Schimmel H., Wang L., Panteghini M., IFCC Working Group on Standardization of Troponin I (2010). Standardisation of cardiac troponin I measurement: Past and present. Pathology.

[63] Panteghini M., Bunk D.M., Christenson R.H., Katrukha A., Porter R.A., Schimmel H., Wang L., Tate J.R., IFCC Working Group on Standardization of Troponin I (2008). Standardization of troponin I measurements: An update. Clin. Chem. Lab. Med..

[64] Jarolim P. (2015). High sensitivity cardiac troponin assays in the clinical laboratories. Clin. Chem. Lab. Med..

[65] Katrukha I.A., Kogan A.E., Vylegzhanina A.V., Kharitonov A.V., Tamm N.N., Filatov V.L., Bereznikova A.V., Koshkina E.V., Katrukha A.G. (2018). Full-Size Cardiac Troponin I and Its Proteolytic Fragments in Blood of Patients with Acute Myocardial Infarction: Antibody Selection for Assay Development. Clin. Chem..

[66] Streng A.S., de Boer D., van der Velden J., van Dieijen-Visser M.P., Wodzig W.K. (2013). Posttranslational modifications of cardiac troponin T: An overview. J. Mol. Cell Cardiol..

[67] Mirzaii-Dizgah I., Riahi E. (2013). Salivary troponin I as an indicator of myocardial infarction. Indian J. Med. Res..

[68] Bahbah E.I., Noehammer C., Pulverer W., Jung M., Weinhaeusel A. (2021). Salivary biomarkers in cardiovascular disease: An insight into the current evidence. FEBS J..

[69] Abdul Rehman S., Khurshid Z., Hussain Niazi F., Naseem M., Al Waddani H., Sahibzada H.A., Sannam Khan R. (2017). Role of Salivary Biomarkers in Detection of Cardiovascular Diseases (CVD). Proteomes.

[70] Klichowska-Palonka M., Załęska-Chromińska K., Bachanek T. (2015). Possibility of using saliva as a diagnostic test material in cardiovascular diseases. Wiad. Lek..

[71] Pervan P., Svaguša T., Prkačin I., Savuk A., Bakos M., Perkov S. (2017). Urine high sensitive Troponin I measuring in patients with hypertension. Signa Vitae.

[72] Mishra V., Patil R., Khanna V., Tripathi A., Singh V., Pandey S., Chaurasia A. (2018). Evaluation of Salivary Cardiac Troponin-I as Potential Marker for Detection of Acute Myocardial Infarction. J. Clin. Diagn. Res..

[73] Chaulin A.M. (2021). Phosphorylation and Fragmentation of the Cardiac Troponin T: Mechanisms, Role in Pathophysiology and Laboratory Diagnosis. Int. J. Biomed..

[74] Ziebig R., Lun A., Hocher B., Priem F., Altermann C., Asmus G., Kern H., Krause R., Lorenz B., Möbes R. (2003). Renal elimination of troponin T and troponin I. Clin. Chem..

[75] Ellis K., Dreisbach A.W., Lertora J.L. (2001). Plasma elimination of cardiac troponin I in end-stage renal disease. South Med. J..

[76] Hernández-Romero D., Valverde-Vázquez M.D.R., Hernández Del Rincón J.P., Noguera-Velasco J.A., Pérez-Cárceles M.D., Osuna E. (2021). Diagnostic Application of Postmortem Cardiac Troponin I Pericardial Fluid/Serum Ratio in Sudden Cardiac Death. Diagnostics.

[77] Zhu B.L., Ishikawa T., Michiue T., Li D.R., Zhao D., Bessho Y., Kamikodai Y., Tsuda K., Okazaki S., Maeda H. (2007). Postmortem cardiac troponin I and creatine kinase MB levels in the blood and pericardial fluid as markers of myocardial damage in medicolegal autopsy. Leg. Med..

[78] González-Herrera L., Valenzuela A., Ramos V., Blázquez A., Villanueva E. (2016). Cardiac troponin T determination by a highly sensitive assay in postmortem serum and pericardial fluid. Forensic Sci. Med. Pathol..

[79] Maeda H., Michiue T., Zhu B.L., Ishikawa T., Quan L. (2009). Analysis of cardiac troponins and creatine kinase MB in cerebrospinal fluid in medicolegal autopsy cases. Leg. Med..

[80] Wang Q., Michiue T., Ishikawa T., Zhu B.L., Maeda H. (2011). Combined analyses of creatine kinase MB, cardiac troponin I and myoglobin in pericardial and cerebrospinal fluids to investigate myocardial and skeletal muscle injury in medicolegal autopsy cases. Leg. Med..

[81] Chen J.H., Inamori-Kawamoto O., Michiue T., Ikeda S., Ishikawa T., Maeda H. (2015). Cardiac biomarkers in blood, and pericardial and cerebrospinal fluids of forensic autopsy cases: A reassessment with special regard to postmortem interval. Leg. Med..

[82] Sessa F., Esposito M., Messina G., Di Mizio G., Di Nunno N., Salerno M. (2021). Sudden Death in Adults: A Practical Flow Chart for Pathologist Guidance. Healthcare.

[83] Stefanovic V., Loukovaara M. (2005). Amniotic fluid cardiac troponin T in pathological pregnancies with evidence of chronic fetal hypoxia. Croat. Med. J..

[84] Yoshida M., Matsuda H., Yoshinaga Y., Asai K., Kawashima A., Sei K., Horii M., Nakanishi A., Soyama H., Furuya K. (2013). Analysis about the influence on the fetus infected with parvovirus B19 using amniotic erythropoietin and troponin-T. Arch. Gynecol. Obstet..

[85] Blohm M.E., Arndt F., Fröschle G.M., Langenbach N., Sandig J., Vettorazzi E., Mir T.S., Hecher K., Weil J., Kozlik-Feldmann R. (2019). Cardiovascular Biomarkers in Amniotic Fluid, Umbilical Arterial Blood, Umbilical Venous Blood, and Maternal Blood at Delivery, and Their Reference Values for Full-Term, Singleton, Cesarean Deliveries. Front. Pediatr..

[86] Van Mieghem T., Doné E., Gucciardo L., Klaritsch P., Allegaert K., Van Bree R., Lewi L., Deprest J. (2010). Amniotic fluid markers of fetal cardiac dysfunction in twin-to-twin transfusion syndrome. Am. J. Obstet. Gynecol..

[87] Chaulin A.M., Duplyakov D.V. (2021). Cardiac troponins: Current data on the diagnostic value and analytical characteristics of new determination methods. Cor. Vasa..

[88] Kavsak P.A. (2018). Should detectable cardiac troponin concentrations in a healthy population be the only criterion for classifying high-sensitivity cardiac troponin assays?. Clin. Biochem..

[89] Ji M., Moon H.W., Hur M., Yun Y.M. (2016). Determination of high-sensitivity cardiac troponin I 99th percentile upper reference limits in a healthy Korean population. Clin. Biochem..

[90] Tjora S., Hall T.S., Larstorp A.C., Hallen J., Atar D. (2018). Increases in Circulating Cardiac Troponin Are Not Always Associated with Myocardial Cell Death. Clin. Lab..

[91] Jaffe A.S., Wu A.H. (2012). Troponin release--reversible or irreversible injury? Should we care?. Clin. Chem..

[92] Mair J., Lindahl B., Hammarsten O., Müller C., Giannitsis E., Huber K., Möckel M., Plebani M., Thygesen K., Jaffe A.S. (2018). How is cardiac troponin released from injured myocardium?. Eur. Heart J. Acute Cardiovasc. Care.

[93] Hammarsten O., Mair J., Möckel M., Lindahl B., Jaffe A.S. (2018). Possible mechanisms behind cardiac troponin elevations. Biomarkers.

[94] Chaulin A.M., Duplyakov D.V. (2021). Mechanisms of increase and diagnostic role of highly sensitive troponins in arterial hypertension. Ann Cardiol. Angeiol..

[95] Gumprecht J., Domek M., Lip G.Y.H., Shantsila A. (2019). Invited review: Hypertension and atrial fibrillation: Epidemiology, pathophysiology, and implications for management. J. Hum. Hypertens..

[96] Liao X.D., Wang X.H., Jin H.J., Chen L.Y., Chen Q. (2004). Mechanical stretch induces mitochondria-dependent apoptosis in neonatal rat cardiomyocytes and G2/M accumulation in cardiac fibroblasts. Cell Res..

[97] Cheng W.P., Wang B.W., Lo H.M., Shyu K.G. (2015). Mechanical Stretch Induces Apoptosis Regulator TRB3 in Cultured Cardiomyocytes and Volume-Overloaded Heart. PLoS ONE.

[98] Jiang S., Huo D., Wang X., Zhao H., Tan J., Zeng Q., O’Rourke S.T., Sun C. (2017). β-adrenergic Receptor-stimulated Cardiac Myocyte Apoptosis: Role of Cytochrome P450 ω-hydroxylase. J. Cardiovasc. Pharmacol..

[99] Communal C., Colucci W.S. (2005). The control of cardiomyocyte apoptosis via the beta-adrenergic signaling pathways. Arch. Mal. Coeur Vaiss..

[100] Dalal S., Foster C.R., Das B.C., Singh M., Singh K. (2012). Β-adrenergic receptor stimulation induces endoplasmic reticulum stress in adult cardiac myocytes: Role in apoptosis. Mol. Cell Biochem..

[101] Chen Q.M., Tu V.C. (2002). Apoptosis and heart failure: Mechanisms and therapeutic implications. Am. J. Cardiovasc. Drugs..

[102] Kunapuli S., Rosanio S., Schwarz E.R. (2006). “How do cardiomyocytes die?” apoptosis and autophagic cell death in cardiac myocytes. J. Card. Fail..

[103] Ricchiuti V., Apple F.S. (1999). RNA expression of cardiac troponin T isoforms in diseased human skeletal muscle. Clin. Chem..

[104] Ricchiuti V., Voss E.M., Ney A., Odland M., Anderson P.A., Apple F.S. (1998). Cardiac troponin T isoforms expressed in renal diseased skeletal muscle will not cause false-positive results by the second generation cardiac troponin T assay by Boehringer Mannheim. Clin. Chem..

[105] Bergmann O., Bhardwaj R.D., Bernard S., Zdunek S., Barnabé-Heider F., Walsh S., Zupicich J., Alkass K., Buchholz B.A., Druid H. (2009). Evidence for cardiomyocyte renewal in humans. Science.

[106] Bergmann O., Zdunek S., Frisén J., Bernard S., Druid H., Jovinge S. (2012). Cardiomyocyte renewal in humans. Circ. Res..

[107] White H.D. (2011). Pathobiology of troponin elevations: Do elevations occur with myocardial ischemia as well as necrosis?. J. Am. Coll. Cardiol..

[108] Nakada Y., Canseco D.C., Thet S., Abdisalaam S., Asaithamby A., Santos C.X., Shah A.M., Zhang H., Faber J.E., Kinter M.T. (2017). Hypoxia induces heart regeneration in adult mice. Nature.

[109] Lázár E., Sadek H.A., Bergmann O. (2017). Cardiomyocyte renewal in the human heart: Insights from the fall-out. Eur. Heart J..

[110] Foglia M.J., Poss K.D. (2016). Building and re-building the heart by cardiomyocyte proliferation. Development.

[111] Docshin P.M., Karpov A.A., Eyvazova S.D., Puzanov M.V., Kostareva A.A., Galagudza M., Malashicheva A.B. (2018). Activation of Cardiac Stem Cells in Myocardial Infarction. Cell Tissue Biol..

[112] Waring C.D., Vicinanza C., Papalamprou A., Smith A.J., Purushothaman S., Goldspink D.F., Nadal-Ginard B., Torella D., Ellison G.M. (2014). The adult heart responds to increased workload with physiologic hypertrophy, cardiac stem cell activation, and new myocyte formation. Eur. Heart J..

[113] Rovira M., Borràs D.M., Marques I.J., Puig C., Planas J.V. (2018). Physiological Responses to Swimming-Induced Exercise in the Adult Zebrafish Regenerating Heart. Front. Physiol..

[114] Schüttler D., Clauss S., Weckbach L.T., Brunner S. (2019). Molecular Mechanisms of Cardiac Remodeling and Regeneration in Physical Exercise. Cells.

[115] Talman V., Ruskoaho H. (2016). Cardiac fibrosis in myocardial infarction-from repair and remodeling to regeneration. Cell Tissue Res..

[116] Isomi M., Sadahiro T., Ieda M. (2019). Progress and Challenge of Cardiac Regeneration to Treat Heart Failure. J. Cardiol..

[117] Zhang J., Liu D., Zhang M., Zhang Y. (2019). Programmed necrosis in cardiomyocytes: Mitochondria, death receptors and beyond. Br. J. Pharmacol..

[118] Lee Y., Gustafsson A.B. (2009). Role of apoptosis in cardiovascular disease. Apoptosis.

[119] Kyrylkova K., Kyryachenko S., Leid M., Kioussi C. (2012). Detection of apoptosis by TUNEL assay. Methods Mol. Biol..

[120] Zorc-Pleskovic R., Alibegović A., Zorc M., Milutinović A., Radovanović N., Petrović D. (2006). Apoptosis of cardiomyocytes in myocarditis. Folia Biol..

[121] Zhang Q., Yu N., Yu B.T. (2018). MicroRNA-298 regulates apoptosis of cardiomyocytes after myocardial infarction. Eur. Rev. Med. Pharmacol. Sci..

[122] Weil B.R., Young R.F., Shen X., Suzuki G., Qu J., Malhotra S., Canty J.M. (2017). Brief Myocardial Ischemia Produces Cardiac Troponin I Release and Focal Myocyte Apoptosis in the Absence of Pathological Infarction in Swine. JACC Basic Transl. Sci..

[123] Cheng W., Li B., Kajstura J., Li P., Wolin M.S., Sonnenblick E.H., Hintze T.H., Olivetti G., Anversa P. (1995). Stretch-induced programmed myocyte cell death. J. Clin. Investig..

[124] Gherasim L. (2019). Troponins in Heart Failure—A Perpetual Challenge. Maedica.

[125] Aengevaeren V.L., Baggish A.L., Chung E.H., George K., Kleiven Ø., Mingels A.M.A., Ørn S., Shave R.E., Thompson P.D., Eijsvogels T.M.H. (2021). Exercise-Induced Cardiac Troponin Elevations: From Underlying Mechanisms to Clinical Relevance. Circulation.

[126] Park K.C., Gaze D.C., Collinson P.O., Marber M.S. (2017). Cardiac troponins: From myocardial infarction to chronic disease. Cardiovasc. Res..

[127] Weil B.R., Suzuki G., Young R.F., Iyer V., Canty J.M. (2018). Troponin Release and Reversible Left Ventricular Dysfunction after Transient Pressure Overload. J. Am. Coll. Cardiol..

[128] Felker G.M., Fudim M. (2018). Unraveling the Mystery of Troponin Elevation in Heart Failure. J. Am. Coll. Cardiol..

[129] Sanchez O., Planquette B., Wermert D., Marié E., Meyer G. (2008). Embolies pulmonaires graves [Massive pulmonary embolism]. Presse Med..

[130] El-Menyar A., Sathian B., Al-Thani H. (2019). Elevated serum cardiac troponin and mortality in acute pulmonary embolism: Systematic review and meta-analysis. Respir. Med..

[131] Daquarti G., March Vecchio N., Mitrione C.S., Furmento J., Ametrano M.C., Dominguez Pace M.P., Costabel J.P. (2016). High-sensitivity troponin and right ventricular function in acute pulmonary embolism. Am. J. Emerg. Med..

[132] Singh K., Communal C., Sawyer D.B., Colucci W.S. (2000). Adrenergic regulation of myocardial apoptosis. Cardiovasc. Res..

[133] Colucci W.S., Sawyer D.B., Singh K., Communal C. (2000). Adrenergic overload and apoptosis in heart failure: Implications for therapy. J. Card. Fail..

[134] Xiao R.P., Tomhave E.D., Wang D.J., Ji X., Boluyt M.O., Cheng H., Lakatta E.G., Koch W.J. (1998). Age-associated reductions in cardiac beta1- and beta2-adrenergic responses without changes in inhibitory G proteins or receptor kinases. J. Clin. Investig..

[135] Mougenot N., Mika D., Czibik G., Marcos E., Abid S., Houssaini A., Vallin B., Guellich A., Mehel H., Sawaki D. (2019). Cardiac adenylyl cyclase overexpression precipitates and aggravates age-related myocardial dysfunction. Cardiovasc. Res..

[136] de Lucia C., Eguchi A., Koch W.J. (2018). New Insights in Cardiac β-Adrenergic Signaling during Heart Failure and Aging. Front. Pharmacol..

[137] Schwartz P., Piper H.M., Spahr R., Spieckermann P.G. (1984). Ultrastructure of cultured adult myocardial cells during anoxia and reoxygenation. Am. J. Pathol..

[138] Siegmund B., Koop A., Klietz T., Schwartz P., Piper H.M. (1990). Sarcolemmal integrity and metabolic competence of cardiomyocytes under anoxia-reoxygenation. Am. J. Physiol..

[139] Piper H.M., Schwartz P., Spahr R., Hütter J.F., Spieckermann P.G. (1984). Absence of reoxygenation damage in isolated heart cells after anoxic injury. Pflug. Arch..

[140] Chaulin A.M. (2021). Updated information about methods of identification and diagnostic opportunities of cardiac troponins. Riv. Ital. Della Med. Lab..

[141] Aakre K.M., Omland T. (2019). Physical activity, exercise and cardiac troponins: Clinical implications. Prog. Cardiovasc. Dis..

[142] Sheyin O., Davies O., Duan W., Perez X. (2015). The prognostic significance of troponin elevation in patients with sepsis: A meta-analysis. Heart Lung.

[143] Gibler W.B., Gibler C.D., Weinshenker E., Abbottsmith C., Hedges J.R., Barsan W.G., Sperling M., Chen I.W., Embry S., Kereiakes D. (1987). Myoglobin as an early indicator of acute myocardial infarction. Ann. Emerg. Med..

[144] Bhayana V., Henderson A.R. (1995). Biochemical markers of myocardial damage. Clin. Biochem..

[145] Chen Y., Tao Y., Zhang L., Xu W., Zhou X. (2019). Diagnostic and prognostic value of biomarkers in acute myocardial infarction. Postgrad. Med. J..

[146] McDonough J.L., Arrell D.K., Van Eyk J.E. (1999). Troponin I degradation and covalent complex formation accompanies myocardial ischemia/reperfusion injury. Circ. Res..

[147] Feng J., Schaus B.J., Fallavollita J.A., Lee T.C., Canty J.M. (2001). Preload induces troponin I degradation independently of myocardial ischemia. Circulation.

[148] Gao C.Q., Sawicki G., Suarez-Pinzon W.L., Csont T., Wozniak M., Ferdinandy P., Schulz R. (2003). Matrix metalloproteinase-2 mediates cytokine-induced myocardial contractile dysfunction. Cardiovasc. Res..

[149] Lin N.N., Cheng C.C., Lee Y.F., Fu Y.C., Chen J.S., Ho S.P., Chiu Y.T. (2013). Early activation of myocardial matrix metalloproteinases and degradation of cardiac troponin I after experimental subarachnoid hemorrhage. J. Surg. Res..

[150] Parente J.M., Blascke de Mello M.M., Silva P.H.L.D., Omoto A.C.M., Pernomian L., Oliveira I.S., Mahmud Z., Fazan R., Arantes E.C., Schulz R. (2021). MMP inhibition attenuates hypertensive eccentric cardiac hypertrophy and dysfunction by preserving troponin I and dystrophin. Biochem. Pharmacol..

[151] Streng A.S., de Boer D., van Doorn W.P., Kocken J.M., Bekers O., Wodzig W.K. (2016). Cardiac troponin T degradation in serum is catalysed by human thrombin. Biochem. Biophys. Res. Commun..

[152] Katrukha I.A., Kogan A.E., Vylegzhanina A.V., Serebryakova M.V., Koshkina E.V., Bereznikova A.V., Katrukha A.G. (2017). Thrombin-Mediated Degradation of Human Cardiac Troponin T. Clin. Chem..

[153] Bodor G.S. (2017). Cardiac Troponins: Molecules of Many Surprises. Clin. Chem..

[154] Ito K., Date T., Ikegami M., Hongo K., Fujisaki M., Katoh D., Yoshino T., Anzawa R., Nagoshi T., Yamashita S. (2013). An immunohistochemical analysis of tissue thrombin expression in the human atria. PLoS ONE.

[155] Ito K., Hongo K., Date T., Ikegami M., Hano H., Owada M., Morimoto S., Kashiwagi Y., Katoh D., Yoshino T. (2017). Tissue thrombin is associated with the pathogenesis of dilated cardiomyopathy. Int. J. Cardiol..

[156] Matsukura U., Okitani A., Nishimuro T., Kato H. (1981). Mode of degradation of myofibrillar proteins by an endogenous protease, cathepsin L. Biochim. Biophys. Acta.

[157] Peng K., Liu H., Yan B., Meng X.W., Song S.Y., Ji F.H., Xia Z. (2021). Inhibition of cathepsin S attenuates myocardial ischemia/reperfusion injury by suppressing inflammation and apoptosis. J. Cell Physiol..

[158] Hickman P.E., Potter J.M., Aroney C., Koerbin G., Southcott E., Wu A.H., Roberts M.S. (2010). Cardiac troponin may be released by ischemia alone, without necrosis. Clin. Chim. Acta.

[159] Hessel M.H., Atsma D.E., van der Valk E.J., Bax W.H., Schalij M.J., van der Laarse A. (2008). Release of cardiac troponin I from viable cardiomyocytes is mediated by integrin stimulation. Pflug. Arch..

[160] Ross R.S., Borg T.K. (2001). Integrins and the myocardium. Circ. Res..

[161] Khabbaz K.R., Feng J., Boodhwani M., Clements R.T., Bianchi C., Sellke F.W. (2008). Nonischemic myocardial acidosis adversely affects microvascular and myocardial function and triggers apoptosis during cardioplegia. J. Thorac. Cardiovasc. Surg..

[162] Thatte H.S., Rhee J.H., Zagarins S.E., Treanor P.R., Birjiniuk V., Crittenden M.D., Khuri S.F. (2004). Acidosis-induced apoptosis in human and porcine heart. Ann. Thorac. Surg..

[163] Graham R.M., Frazier D.P., Thompson J.W., Haliko S., Li H., Wasserlauf B.J., Spiga M.G., Bishopric N.H., Webster K.A. (2004). A unique pathway of cardiac myocyte death caused by hypoxia-acidosis. J. Exp. Biol..

[164] Wasfy M.M., Hutter A.M., Weiner R.B. (2016). Sudden Cardiac Death in Athletes. Methodist Debakey Cardiovasc. J..

[165] Aune D., Schlesinger S., Hamer M., Norat T., Riboli E. (2020). Physical activity and the risk of sudden cardiac death: A systematic review and meta-analysis of prospective studies. BMC Cardiovasc. Disord..

[166] DeFroda S.F., McDonald C., Myers C., Cruz A.I., Owens B.D., Daniels A.H. (2019). Sudden Cardiac Death in the Adolescent Athlete: History, Diagnosis, and Prevention. Am. J. Med..

[167] Sollazzo F., Palmieri V., Gervasi S.F., Cuccaro F., Modica G., Narducci M.L., Pelargonio G., Zeppilli P., Bianco M. (2021). Sudden Cardiac Death in Athletes in Italy during 2019: Internet-Based Epidemiological Research. Medicina.

[168] Klinkenberg L.J., Luyten P., van der Linden N., Urgel K., Snijders D.P., Knackstedt C., Dennert R., Kietselaer B.L., Mingels A.M., Cardinaels E.P. (2016). Cardiac Troponin T and I Release After a 30-km Run. Am. J. Cardiol..

[169] Martínez-Navarro I., Sánchez-Gómez J., Sanmiguel D., Collado E., Hernando B., Panizo N., Hernando C. (2020). Immediate and 24-h post-marathon cardiac troponin T is associated with relative exercise intensity. Eur. J. Appl. Physiol..

[170] Marshall L., Lee K.K., Stewart S.D., Wild A., Fujisawa T., Ferry A.V., Stables C.L., Lithgow H., Chapman A.R., Anand A. (2020). Effect of Exercise Intensity and Duration on Cardiac Troponin Release. Circulation.

[171] O’Hanlon R., Wilson M., Wage R., Smith G., Alpendurada F.D., Wong J., Dahl A., Oxborough D., Godfrey R., Sharma S. (2010). Troponin release following endurance exercise: Is inflammation the cause? A cardiovascular magnetic resonance study. J. Cardiovasc. Magn. Reson..

[172] Lazzarino A.I., Hamer M., Gaze D., Collinson P., Steptoe A. (2013). The association between cortisol response to mental stress and high-sensitivity cardiac troponin T plasma concentration in healthy adults. J. Am. Coll. Cardiol..

[173] Eggers K.M. (2013). Mental stress and cardiac troponin: Keep calm and carry on?. J. Am. Coll. Cardiol..

[174] Yamaji M., Tsutamoto T., Kawahara C., Nishiyama K., Yamamoto T., Fujii M., Horie M. (2009). Serum cortisol as a useful predictor of cardiac events in patients with chronic heart failure: The impact of oxidative stress. Circ. Heart Fail..

[175] Iwaszczuk P., Łosiak W., Szczeklik W., Musiałek P. (2021). Patient periprocedural stress in cardiovascular medicine: Friend or foe?. Postępy Kardiol. Interwencyjnej.

[176] Bakay M., Zhao P., Chen J., Hoffman E.P. (2002). A web-accessible complete transcriptome of normal human and DMD muscle. Neuromuscul. Disord..

[177] Messner B., Baum H., Fischer P., Quasthoff S., Neumeier D. (2000). Expression of messenger RNA of the cardiac isoforms of troponin T and I in myopathic skeletal muscle. Am. J. Clin. Pathol..

[178] Rusakov D.Y., Yamshcikov N.V., Tulayeva O.N., Suvorova L.A., Metlenko O.I. (2015). Histogenesis and pecularities of structural organization of the cardiac muscle tissue un the walls of human caval and pulmonary veins. Morphology.

[179] Rusakov D.Y., Vologdina N.N., Tulayeva O.N. (2015). The development of striated cardiac muscle tissue in the walls of the caval and pulmonary veins. J. Anat. Histopathol..

[180] Bodor G.S., Porterfield D., Voss E.M., Smith S., Apple F.S. (1995). Cardiac troponin-I is not expressed in fetal and healthy or diseased adult human skeletal muscle tissue. Clin. Chem..

[181] Hammerer-Lercher A., Erlacher P., Bittner R., Korinthenberg R., Skladal D., Sorichter S., Sperl W., Puschendorf B., Mair J. (2001). Clinical and experimental results on cardiac troponin expression in Duchenne muscular dystrophy. Clin. Chem..

[182] Schmid J., Liesinger L., Birner-Gruenberger R., Stojakovic T., Scharnagl H., Dieplinger B., Asslaber M., Radl R., Beer M., Polacin M. (2018). Elevated Cardiac Troponin T in Patients With Skeletal Myopathies. J. Am. Coll. Cardiol..

[183] Anderson P.A., Greig A., Mark T.M., Malouf N.N., Oakeley A.E., Ungerleider R.M., Allen P.D., Kay B.K. (1995). Molecular basis of human cardiac troponin T isoforms expressed in the developing, adult, and failing heart. Circ. Res..

[184] Bates K.J., Hall E.M., Fahie-Wilson M.N., Kindler H., Bailey C., Lythall D., Lamb E.J. (2010). Circulating immunoreactive cardiac troponin forms determined by gel filtration chromatography after acute myocardial infarction. Clin. Chem..

[185] Maekawa A., Lee J.K., Nagaya T., Kamiya K., Yasui K., Horiba M., Miwa K., Uzzaman M., Maki M., Ueda Y. (2003). Overexpression of calpastatin by gene transfer prevents troponin I degradation and ameliorates contractile dysfunction in rat hearts subjected to ischemia/reperfusion. J. Mol. Cell Cardiol..

[186] Zahran S., Figueiredo V.P., Graham M.M., Schulz R., Hwang P.M. (2018). Proteolytic Digestion of Serum Cardiac Troponin I as Marker of Ischemic Severity. J. Appl. Lab. Med..

[187] Vylegzhanina A.V., Kogan A.E., Katrukha I.A., Koshkina E.V., Bereznikova A.V., Filatov V.L., Bloshchitsyna M.N., Bogomolova A.P., Katrukha A.G. (2019). Full-Size and Partially Truncated Cardiac Troponin Complexes in the Blood of Patients with Acute Myocardial Infarction. Clin. Chem..

[188] Katus H.A., Remppis A., Looser S., Hallermeier K., Scheffold T., Kubler W. (1989). Enzyme linked immune assay of cardiac troponin T for the detection of acute myocardial infarction in patients. J. Mol. Cell Cardiol..

[189] Labugger R., Organ L., Collier C., Atar D., Van Eyk J.E. (2000). Extensive troponin I and T modification detected in serum from patients with acute myocardial infarction. Circulation.

[190] Gaze D.C., Collinson P.O. (2008). Multiple molecular forms of circulating cardiac troponin: Analytical and clinical significance. Ann. Clin. Biochem..

[191] Katrukha A.G., Bereznikova A.V., Esakova T.V., Pettersson K., Lövgren T., Severina M.E., Pulkki K., Vuopio-Pulkki L.M., Gusev N.B. (1997). Troponin I is released in bloodstream of patients with acute myocardial infarction not in free form but as complex. Clin. Chem..

[192] Bodor G.S., Oakeley A.E., Allen P.D., Crimmins D.L., Ladenson J.H., Anderson P.A. (1997). Troponin I phosphorylation in the normal and failing adult human heart. Circulation.

[193] Hayashi T., Notkins A.L. (1994). Clearance of LDH-5 from the circulation of inbred mice correlates with binding to macrophages. Int. J. Exp. Pathol..

[194] Prabhudas M., Bowdish D., Drickamer K., Febbraio M., Herz J., Kobzik L., Krieger M., Loike J., Means T.K., Moestrup S.K. (2014). Standardizing scavenger receptor nomenclature. J. Immunol..

[195] De Zoysa J.R. (2004). Cardiac troponins and renal disease. Nephrology.

[196] Dubin R.F., Li Y., He J., Jaar B.G., Kallem R., Lash J.P., Makos G., Rosas S.E., Soliman E.Z., Townsend R.R. (2013). Predictors of high sensitivity cardiac troponin T in chronic kidney disease patients: A cross-sectional study in the chronic renal insufficiency cohort (CRIC). BMC Nephrol..

[197] Di Lullo L., Barbera V., Santoboni A., Bellasi A., Cozzolino M., De Pascalis A., Rivera R., Balducci A., Russo D., Ronco C. (2015). Malattia renale cronica e sindrome coronarica acuta: Il ruolo della troponina [Troponins and chronic kidney disease]. G. Ital. Nefrol..

[198] Han X., Zhang S., Chen Z., Adhikari B.K., Zhang Y., Zhang J., Sun J., Wang Y. (2020). Cardiac biomarkers of heart failure in chronic kidney disease. Clin. Chim Acta..

[199] Wilhelm J., Hettwer S., Schuermann M., Bagger S., Gerhardt F., Mundt S., Muschik S., Zimmermann J., Amoury M., Ebelt H. (2014). Elevated troponin in septic patients in the emergency department: Frequency, causes, and prognostic implications. Clin. Res. Cardiol..

[200] Røsjø H., Varpula M., Hagve T.A., Karlsson S., Ruokonen E., Pettilä V., Omland T., FINNSEPSIS Study Group (2011). Circulating high sensitivity troponin T in severe sepsis and septic shock: Distribution, associated factors, and relation to outcome. Intensive Care Med..

[201] Daly M., Long B., Koyfman A., Lentz S. (2020). Identifying cardiogenic shock in the emergency department. Am. J. Emerg. Med..

[202] Muslimovic A., Fridén V., Tenstad O., Starnberg K., Nyström S., Wesén E., Esbjörner E.K., Granholm K., Lindahl B., Hammarsten O. (2020). The Liver and Kidneys mediate clearance of cardiac troponin in the rat. Sci. Rep..

[203] Fridén V., Starnberg K., Muslimovic A., Ricksten S.E., Bjurman C., Forsgard N., Wickman A., Hammarsten O. (2017). Clearance of cardiac troponin T with and without kidney function. Clin. Biochem..

[204] Kavsak P.A., Worster A., Shortt C., Ma J., Clayton N., Sherbino J., Hill S.A., McQueen M., Griffith L.E., Mehta S.R. (2018). Performance of high-sensitivity cardiac troponin in the emergency department for myocardial infarction and a composite cardiac outcome across different estimated glomerular filtration rates. Clin. Chim. Acta.

[205] Patke A., Young M.W., Axelrod S. (2020). Molecular mechanisms and physiological importance of circadian rhythms. Nat. Rev. Mol. Cell Biol..

[206] Cribbet M.R., Logan R.W., Edwards M.D., Hanlon E., Bien Peek C., Stubblefield J.J., Vasudevan S., Ritchey F., Frank E. (2016). Circadian rhythms and metabolism: From the brain to the gut and back again. Ann. N. Y. Acad. Sci..

[207] Thosar S.S., Butler M.P., Shea S.A. (2018). Role of the circadian system in cardiovascular disease. J. Clin. Investig..

[208] Klinkenberg L.J.J., Wildi K., van der Linden N., Kouw I.W.K., Niens M., Twerenbold R., Gimenez M.R., Puelacher C., Neuhaus J.D., Hillinger P. (2016). Diurnal rhythm of cardiac troponin: Consequences for the diagnosis of acute myocardial infarction. Clin. Chem..

[209] van der Linden N., Cornelis T., Klinkenberg L.J.J., Kimenai D.M., Hilderink J.M., Litjens E.J.R. (2016). Strong diurnal rhythm of troponin T, but not troponin I, in a patient with renal dysfunction. Int. J. Cardiol..

[210] Chaulin A.M., Duplyakov D.V. (2021). Cardiac troponins in hypertension: Mechanisms of increase and diagnostic value. Arter. Gipertenz. Arter. Hypertens..

[211] Tofler G.H., Brezinski D., Schafer A.I., Czeisler C.A., Rutherford J.D., Willich S.N., Gleason R.E., Williams G.H., Muller J.E. (1987). Concurrent morning increase in platelet aggregability and the risk of myocardial infarction and sudden cardiac death. N. Engl. J. Med..

[212] Chaulin A.M., Duplyakov D.V. (2021). Comorbidity in chronic obstructive pulmonary disease and cardiovascular disease. Cardiovasc. Ther. Prev..

[213] Chaulin A.M., Duplyakov D.V. (2021). Microrna: The role in the pathophysiology of atrial fibrillation and potential use as a biomarker. Bull. Sib. Med..

[214] Chaulin A.M., Duplyakov D.V. (2021). Environmental factors and cardiovascular diseases. Hyg. Sanit..

[215] Panza J.A., Epstein S.E., Quyyumi A.A. (1991). Circadian variation in vascular tone and its relation to alpha-sympathetic vasoconstrictor activity. N. Engl. J. Med..

[216] Tsareva Y.O., Mayskova E.A., Fedotov E.A., Shvarts Y.G. (2019). Circadian rhythms of thyroid hormones in patients with ischemic heart disease, arterial hypertension, and atrial fibrillation. Kardiologiia.

[217] Chaulin A.M., Grigorieva J.V., Suvorova G.N., Duplyakov D.V. (2021). Experimental Modeling of Hypothyroidism: Principles, Methods, Several Advanced Research Directions in Cardiology. Russ. Open Med. J..

[218] Chaulin A.M. (2021). Diagnostic value of highly sensitive cardiac troponins and mechanisms of their increase in serum and urine in arterial hypertension. Riv. Ital. Med. Lab..

[219] Suárez-Barrientos A., López-Romero P., Vivas D., Castro-Ferreira F., Núñez-Gil I., Franco E., Ruiz-Mateos B., García-Rubira J.C., Fernández-Ortiz A., Macaya C. (2011). Circadian variations of infarct size in acute myocardial infarction. Heart.

[220] Arroyo Úcar E., Dominguez-Rodriguez A., Abreu-Gonzalez P. (2012). Influencia de la variabilidad diurna en el tamaño del infarto agudo de miocardio [Influence of diurnal variation in the size of acute myocardial infarction]. Med. Intensiva.

[221] Seneviratna A., Lim G.H., Devi A., Carvalho L.P., Chua T., Koh T.H., Tan H.C., Foo D., Tong K.L., Ong H.Y. (2015). Circadian Dependence of Infarct Size and Acute Heart Failure in ST Elevation Myocardial Infarction. PLoS ONE.

[222] Chaulin A.M., Duplyakov D.V. (2022). Cardioprotective Strategies for Doxorubicin-induced Cardiotoxicity: Present and Future. Ration. Pharmacother. Cardiol..

[223] Manfredini R., Boari B., Bressan S., Gallerani M., Salmi R., Portaluppi F., Mehta R.H. (2004). Influence of circadian rhythm on mortality after myocardial infarction: Data from a prospective cohort of emergency calls. Am. J. Emerg. Med..

[224] Fournier S., Puricel S., Morawiec B., Eeckhout E., Mangiacapra F., Trana C., Tapponnier M., Iglesias J.F., Michiels V., Stauffer J.C. (2014). Relationship between time of day and periprocedural myocardial infarction after elective angioplasty. Chronobiol. Int..

[225] Chaulin A.M. (2021). Cardiac Troponins Metabolism: From Biochemical Mechanisms to Clinical Practice (Literature Review). Int. J. Mol. Sci..

[226] Fournier S., Muller O. (2015). Commentary “Recent advances in circadian rhythms in cardiovascular system”. Front. Pharmacol..

